# ﻿Four new *Parasterope* (Ostracoda, Myodocopina) from the Northwest Pacific and their phylogeny based on 16S rRNA

**DOI:** 10.3897/zookeys.1095.77996

**Published:** 2022-04-13

**Authors:** Huyen T. M. Pham, Ivana Karanovic

**Affiliations:** 1 Department of Life Science, Research Institute for Convergence of Basic Science, College of Natural Sciences, Hanyang University, Seoul, 04763, Republic of Korea Hanyang University Seoul Republic of Korea; 2 Institute for Marine and Antarctic Studies, University of Tasmania, Private Bag 49, 7001, Hobart, Tasmania, Australia University of Tasmania Hobart Australia

**Keywords:** Crustacea, East Asia, marine benthos, new species, taxonomic key

## Abstract

*Parasterope* Kornicker, 1975 is a marine ostracod genus with 49 species described so far, which makes it the most diverse representative of the subfamily Cylindroleberidinae, as well as the entire family Cylindroleberididae. Despite its global distribution no species are reported from South Korea. Three new species collected from the Korean coast of the Sea of Japan (*Parasteropebusanensis***sp. nov.**, *P.singula***sp. nov.**, and *P.sohi***sp. nov.**), and one from the Japanese coast of the Pacific Ocean (*P.sagami***sp. nov.**) are described. A taxonomic key to all named species from East Asia is provided. A phylogenetic tree is reconstructed based on partial 16S rRNA sequences of the four new species and other Cylindroleberidinae available from GenBank. Monophyly of *Parasterope* is supported by high posterior probabilities, but the phylogenetic analyses also indicate that some of the GenBank data attributed to this genus are probably misidentifications. A map of distribution and a checklist of all described *Parasterope* species are also provided.

## ﻿Introduction

**Figure 1.**
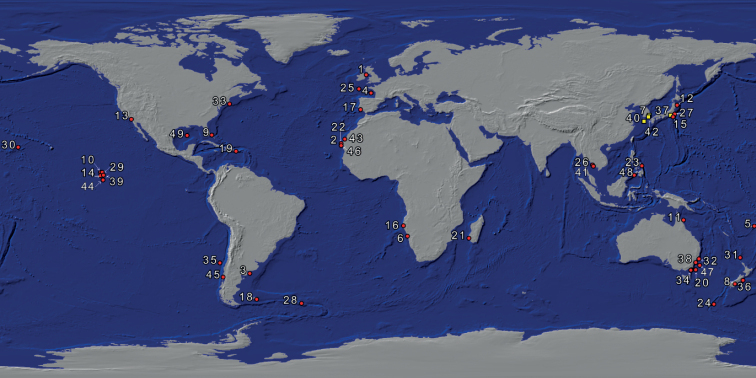
Geographic distribution of *Parasterope* Kornicker, 1975 based on the species’ type localities: red circle indicates previously known species; yellow square indicates species from this study. All the numbers follow the order of species on the checklist (Suppl. material 1).

Cylindroleberididae is one of the largest myodocopid families with remarkable morphological diversity. It accounts for 225 species classified in 33 genera and the following four subfamilies: Cylindroleberidinae, Cyclasteropinae, Asteropteroninae, and Macroasteropteroninae. The primary defining feature of the family is the presence of seven or eight leaf-like pairs of gills at the posterior end of the body, the presence of a “baleen-comb” on the maxilla and the fifth limb, a mandible with a sword-shaped coxal endite, and a hatchet shaped sixth limb (Müller 1906; Poulsen 1965). The family has a worldwide marine distribution and can be found from shallow waters to depths of more than 4570 m (Kornicker 1975). The most speciose of all cylindroleberid genera is *Parasterope* Kornicker, 1975 (of the subfamily Cylindroleberidinae) with 47 species and subspecies currently listed on the World Ostracod Database (Brandão et al. 2022), although six of the listed species are considered synonyms. *Parasterope* representatives have the following combination of characters: first antenna has 0+6 (proximal+distal filament configuration) sensory bristles and the d-bristle is minute or absent; mandible has an e-bristle; and the posterior infold has no ridges between list and the edge of the valve (Poulsen 1965; Kornicker 1975). Species can be found in shallow waters, such as sandy mud flats near mangrove area (i.e., *P.zamboangae* Kornicker, 1970), to abyssal depths of 4303 m (i.e., *P.styx* Kornicker, 1975). The genus has a global distribution (Fig. 1). Of all species recognized so far, only six have been recorded from East Asia: three from Japan (*P.jenseni* Poulsen, 1965; *P.obesa* Poulsen, 1965; and *P.hirutai* Chavtur, 1983), two from the Philippines (*P.zamboangae* Kornicker, 1970 and *P.mckenziei* Kornicker, 1970), and one from Thailand (*P.nana* Poulsen, 1965) (Chavtur 1983; Poulsen 1965; Kornicker 1970).

The cylindroleberid fauna of East Asia is generally poorly known and mostly consists of species records without many details on their morphology. For example, a systematic study of the Korean ostracods as indicators of water pollution lists nine Cylindroleberidae, all left in the open nomenclature (Lee et al. 2000). Similarly, Tanaka and Ohtsuka (2015) reported eight cylindroleberids from Japan, belonging to six genera, of which only three were named: Cyclasteropecf.hilgendorfii (Müller, 1890), *Leuroleberissurugaensis* Hiruta, 1982, and Tetraleberiscf.brevis (Müller, 1890). Most recently, Pham et al. (2021) published the first taxonomic record of the entire family from Korea and described five species, of which one was proposed as a member of a new genus, *Toyoshioleberis* Pham, Jöst & Karanovic, 2021, one was a new record of *Xenoleberisyamadai* (Hiruta, 1979) from Korea, and another two were new to science, *X.parvus* Pham, Jöst & Karanovic, 2021 and *X.tanakai* Pham, Jöst & Karanovic, 2021.

In the present paper, we describe four new species belonging to *Parasterope* that have been collected from seas around Korea and Japan. To facilitate their further identification, we provide a taxonomic key to all East Asian species. In addition, partial 16S rRNA sequences of all new species and partial 18S rRNA sequences of only three new species were successfully amplified. Currently, there are two 16S rRNA sequences, five 18S rRNA sequences, and eleven 28S rRNA sequences available on GenBank (Benson et al. 2015) attributed to *Parasterope*. They belong to three named species and two in the open nomenclature. These GenBank sequences were used in previous studies that aimed to resolve the relationships between Cylindroleberididae and other myodocopid families (Yamaguchi and Endo 2003; Oakley 2005; Syme and Oakley 2012), but the phylogenetic relationship between *Parasterope* species has never been specifically mentioned. In general, phylogenetic relationships between the four cylindroleberid subfamilies is still not resolved despite many attempts to do so by using morphology only (Kornicker 1981), or a combination of morphological and molecular data (Syme 2007; Syme and Oakley 2012). The most recent phylogenetic tree constructed based on a concatenated data set with a combination of 16S, 18S, and 28S markers (Pham et al. 2021) provided little resolution to the problem of polyphyletic nature of the subfamily Cyclasteropinae. In addition, many of the genera on all phylogenetic trees reconstructed so far seem to be paraphyletic or polyphyletic. Resolving intragenic relationship could be a step towards understanding phylogeny on the higher systematic levels. Here we use newly obtained 16S rRNA sequences in combination with those already available on GenBank to reconstruct phylogenetic relationship between *Parasterope* species and the position of the genus within the subfamily Cylindroleberidane. The marker 16S is proven to be suitable for evolutionary studies at lower taxonomic levels for many animal groups (Bridge et al. 1995; Collins 2000; Collins et al. 2006; Govindarajan et al. 2006), including ostracods (see Pham et al. 2020). We also provide a list of all known *Parasterope* species with currently recognized synonyms (Suppl. material 1).

## ﻿Materials and methods

Samples from Japan were collected from Sagami Bay during the 6^th^JAMBIO (Japanese Association for Marine Biology) project, on 13 February 2015 with a dredge from shallow waters (130–241m) (Nakano et al. 2015). Samples were fixed in 99% ethanol on site and fractioned with 500 μm and 300 μm sieves. From the sea around the two Korean Islands, Maemul and Chuja, samples were collected with a dredge net from a boat. Collecting methods were described in Karanovic and Soh (2015). Ostracods were also collected from Minrack harbor (Busan), by diving at 30 m, by members of MABIK (Marine Biodiversity Institute of Korea). Specimens were dissected and soft parts mounted on slides in CMC-10 Mounting Media (Masters Company, Inc.), while carapaces were kept on the SEM stubs. All drawings were prepared using a drawing tube attached to the Olympus BX51 microscope. For observations under the scanning electron microscope (**SEM**) carapaces were coated with platinum. SEM photographs were taken at Eulji University (Korea) with the Hitachi S-4700 electron microscope. All specimens are deposited in the invertebrate collection of the National Institute of the Biological Resources (**NIBR**) in South Korea. The distribution map has been created with the Map Creator Private version 2.0.

The extraction followed the HotSHOT method described in Williams et al. (1992). All PCR reactions were carried out in 25 μl volume containing: 5 μl of diluted DNA template, 1 μl of each forward and reverse primer, 15 μl ultra-distilled water and 5 μl AccuPower® PCR Premix (Bioneer Inc.). Fragments of 16S were amplified using the specific primer pairs 16S MYOF1: CCGGTTTGAACTCAGATCAC; 16S MYOF2: TCAAATCATGTTGARATTWAATGG; 16S MYOR1: GTYTTTTAATTGGRGACTGG; 16S MYOR2: TAAACGGCTGCGGTATYYTG. The primer pairs of Cylind_18S_F (5´-GGGAGCATTTATTAGACCAAAACC-3´) and Cylind_18S_R (5´-TTCCCGTGTTGAGTCAAATTAAGCC-3’) were used for the amplification of the 18S rRNA gene fragment. All PCR reactions run in a TaKaRa PCR Thermal Cycler Dice, using 3.5 μl DNA template, 17 μl ultra-distilled water, 5 μl AccuPower® PCR Premix (Bioneer Inc., Korea), and 1 μl of forward and backward primer each. PCR setting consisted of initial denaturation at 94 °C for 5 min, 35 cycles of denaturation for 30 s at 94 °C, annealing for 30 s at 48 °C for 16S/ 1 min at 50 °C for 18S, extension at 72 °C for 10 min before decreasing to 4 °C at the end. PCR products were electrophoresed (for 20 min at 100 V) on 1% agarose gels (0.5X TAE buffer dyed with GelRed® Nucleic Acid Gel Stain) to determine the presence of target DNA bands. PCR products were purified for sequencing by ethanol precipitation and neutralized by Sodium acetate (pH 5.5). Sequencing reactions were run for both strands to confirm sequence reliability using the Sanger method for dideoxy sequencing (Bionic Inc., Seoul, Korea). All 16S rRNA and 18S rRNA sequences have been deposited in GenBank (Table 1).

**Table 1.** List of 16S sequences used for phylogenetic analysis. ^1^Syme and Oakley (2012); ^2^Wakayama and Abe (2006); ^3^Pham et al. (2021); ^4^Oakley and Cunningham (2002); numbers in bold indicate sequences from this study.

Genus	Species	GenBank number	
		16S	18S
*Bathyleberis*	*B.oculata*	EU587251 ^1^	EU591814 ^1^
	*C.marranyin*		EU587243 ^1^
*Cylindroleberis*	*Cylindroleberis* J57069	EU587253 ^1^	EU587244 ^1^
	*Cylindroleberis* NW-2004	AY624729 ^2^	
UnID	Cylindroleberididae J57076	EU587257 ^1^	
*Parasterope*	*P.busanensis* sp. nov. 24_6	**OK048681**	**OK048719**
	*P.busanensis* sp. nov. 24_7	**OK048682**	**OK048720**
	*P.gamurru* J53224	EU587255 ^1^	EU591819 ^1^
	*P.pollex*		AF363309 ^4^
	*P.sagami* sp. nov. 26_9	**OK048683**	**OK048721**
	*P.sagami* sp. nov. 27_0	**OK048684**	**OK048722**
	*P.singula* sp. nov. 1_1	**OK048686**	**OK048723**
	*P.singula* sp. nov. 1_2	**OK048687**	
	*P.sohi* sp. nov.	**OK048685**	
	*P.styx*		EU587236 ^1^
	*Parasterope* J57072*		EU587247 ^1^
	*Parasterope* NW-2004	AY624728 ^2^	
*Postasterope*	*P.barensi* J57079	EU587258 ^1^	EU587248 ^1^
	*P.corrugata*	EU587259 ^1^	EU591816 ^1^
*Synasterope*	*Synasterope* J57066	EU587252 ^1^	EU587250 ^1^
	*Synasterope* J57067		EU591815 ^1^
*Toyoshioleberis*	*T.magnabucca*	MW534153 ^3^	MZ092883 ^3^
*Xenoleberis*	*X.parvus*	MW534150 ^3^	
	*X.pacifica* 38	MW534151 ^3^	MZ092881 ^3^
	*X.pacifica* 39	MW534152 ^3^	MZ092882 ^3^
	*X.pacifica* 275	MW534140 ^3^	MZ092883 ^3^
	*X.tanakai* 266	MW534141 ^3^	
	*X.tanakai* 284	MW534142 ^3^	MZ092884 ^3^
	*X.yamadai* 272	MW534143 ^3^	MZ092879 ^3^
	*X.yamadai* 273	MW534144 ^3^	MZ092880 ^3^
	*X.yamadai* 14	MW534145 ^3^	MZ092878 ^3^
	*X.yamadai* 30	MW534147 ^3^	

Forward and Reverse strands were visually compared and checked for signal quality and low-resolution sites using FinchTV (version 1.4.0) (Geospiza, Inc., Seattle, WA, USA; http://www.geospiza.com). The strands were aligned with MAFFT v.7 127b (Katoh et al. 2019) using the EINS-i algorithm. All sequences used in the phylogenetic analysis are in the Table 1. The best fit evolutionary model was used based on the Akaike Information Criterion (AIC) as implemented in ModelFinder (Kalyaanamoorthy et al. 2017). Bayesian Inference, implemented in BEAST v2.6.4 (Bouckaert et al. 2014), was used to estimate phylogenetic relationships. Settings included the best fit evolutionary model with four gamma categories, a strict molecular clock, gamma shape alpha 0.645, and 0.3583 in proportion of invariable site. The analysis run for 10,000,000 generations, sampling every 1,000 generations. Software Tracer (Rambaut et al. 2018) was used to visualize results of the BEAST analyses and FigTree v1.4.3 (Rambaut 2010) for tree visualization. Molecular pairwise distances were calculated as uncorrected p-distances (Kimura 1981) and intrageneric distances were calculated as within group mean in MEGA 7 (Kumar et al. 2016).

### ﻿Abbreviations used in text and figures

**A1** first antenna;

**A2** second antenna;

**F** Furca;

**L5** fifth limb;

**L6** sixth limb;

**L7** seventh limb;

**Md** mandibula;

**Mxl** maxillula;

**NIBR** National Institute of Biodiversity Research;

**SEM** scanning electron microscope.

## ﻿Results

### ﻿Systematics

**Phylum Arthropoda Latreille, 1829**

**Subphylum Crustacea Brünnich, 1772**

**Class Ostracoda Latreille, 1802**

**Subclass Myodocopa Sars, 1866**

**Order Myodocopida Sars, 1866**

**Family Cylindroleberididae Müller, 1906**

**Genus *Parasterope* Kornicker, 1994**

### ﻿Key to *Parasterope* from East and Southeast Asia

**Table:** 

1	Mandible, dorsal margin of basale with mid-bristle	**2**
–	Mandible, dorsal margin of basale without mid-bristle	**3**
2	6^th^ limb, ventral and postero-ventral margin without plumose bristles	***P.nana* Poulsen, 1965**
–	6^th^ limb, ventral margin of end-joint with 11 or 12 plumose bristles	***P.zamboangae* Kornicker, 1970**
3	Adult female Md with 3 long terminal setae next to exopodite	***P.sagami* sp. nov.**
–	Adult female Md with 2 long terminal setae next to exopodite	4
4	7^th^ limb with 4 bristles (2 on each side) on the terminal segment	***P.singula* sp. nov.**
–	7^th^ limb with 6 bristles (3 on each side) on the terminal segment	**5**
5	Mandibular basale with 6 spinous end bristles	***P.sohi* sp. nov.**
–	Mandibular basale with 3 or 4 spinous end bristles	**6**
6	Mandibular basale spinous and with cluster of spines near middle	***P.mckenziei* Kornicker, 1970**
–	Mandibular basale with no hair on broad surface	**7**
7	First antenna 8^th^ joint with small spine-like d-bristle	***P.hirutai* Chavtur, 1983**
–	First antenna 8^th^ joint without d-bristle	**8**
8	An open row of ~ 7–15 long medial bristles between the posterior ridge and the shell margin	***P.obesa* Poulsen, 1965**
–	No bristles or only short, scattered bristles between the posterior ridge and the shell margin	**9**
9	2^nd^ joint of first antenna without lateral bristle; second antenna protopodite with a row of spines on the ventral margin	***P.jenseni* Poulsen, 1965**
–	2^nd^ joint of first antenna with lateral bristle; second antenna protopodite without spine on the ventral margin	***P.busanensis* sp. nov.**

#### ﻿ Parasterope busanensis sp. nov.

Taxon classificationAnimaliaMyodocopidaCylindroleberididae

2865C2CC-5A01-5C82-A571-07458A567434

http://zoobank.org/E03199FB-A217-4451-B924-45A64817C83B

Figs 2 , 3 , 4

##### Specimens examined.

***Holotype*** female dissected on one slide, shells on SEM stub (NIBR IV 0000879898_1). ***Paratypes***: three males and one female dissected on one slide, shells on SEM stub (NIBR IV 0000879898_2;3;4 &5). All from the type locality: South Korea, Busan, Min-rack harbor (35°09'11.9"N, 129°07'38.4"E), collected by the Marin Biodiversity Institute of Korea (MABIK) on 11 April 2019.

##### Etymology.

This species is named in reference to the type locality. It is used as an adjective for a place, with the Latin suffix -*ensis*.

##### Diagnosis.

Surface of the shell mostly smooth. Posterior infold of the carapace with a broad shelf. Dorsal margin rounded in both female and male. Posterior end clearly wider than anterior. Lateral eye well developed with black pigmented ommatidia. A1 8-segmented without d-bristle on the 8^th^ joint. Uropodal lamellae with nine claws.

##### Description.

**Figure 2.**
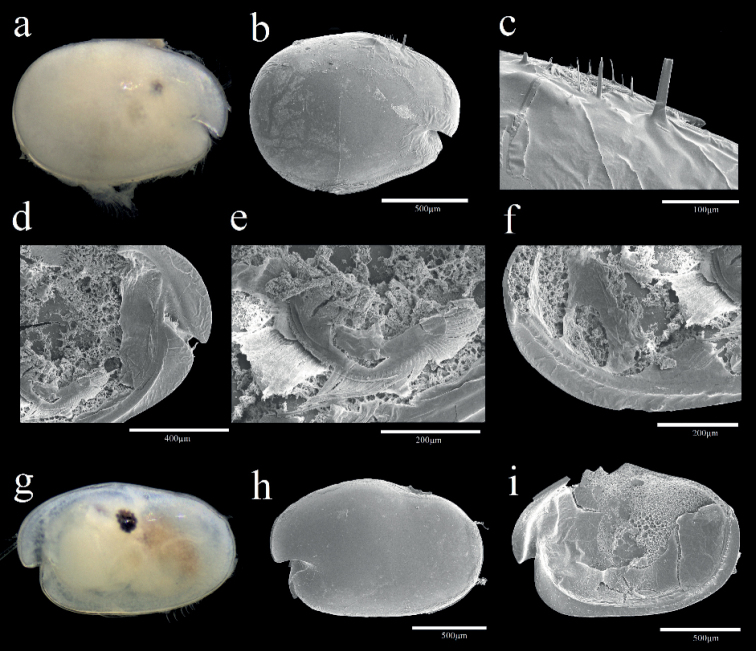
Light microscopy and scanning electron microscopy images of *Parasteropebusanensis* sp. nov. **a–f** holotype female adult: **a** External view **b** Lateral view from left valve **c** Spines on dorsal margin **d** Internal view of anterior part **e**L5 ventral section **f** Internal view of posterior part **g, h** paratype male adult: **g** External view **h** Lateral view from right valve **i** Internal view of left valve.

**Female.** Shell (Fig. 2a–f) Carapace oval, broadening at posterior, greatest height near middle, carapace length 1.25 mm, height 0.94 mm. Nine small horn-like spines near dorsal margin. Posterior infold between broad list and valve margin without setae.

**Figure 3.**
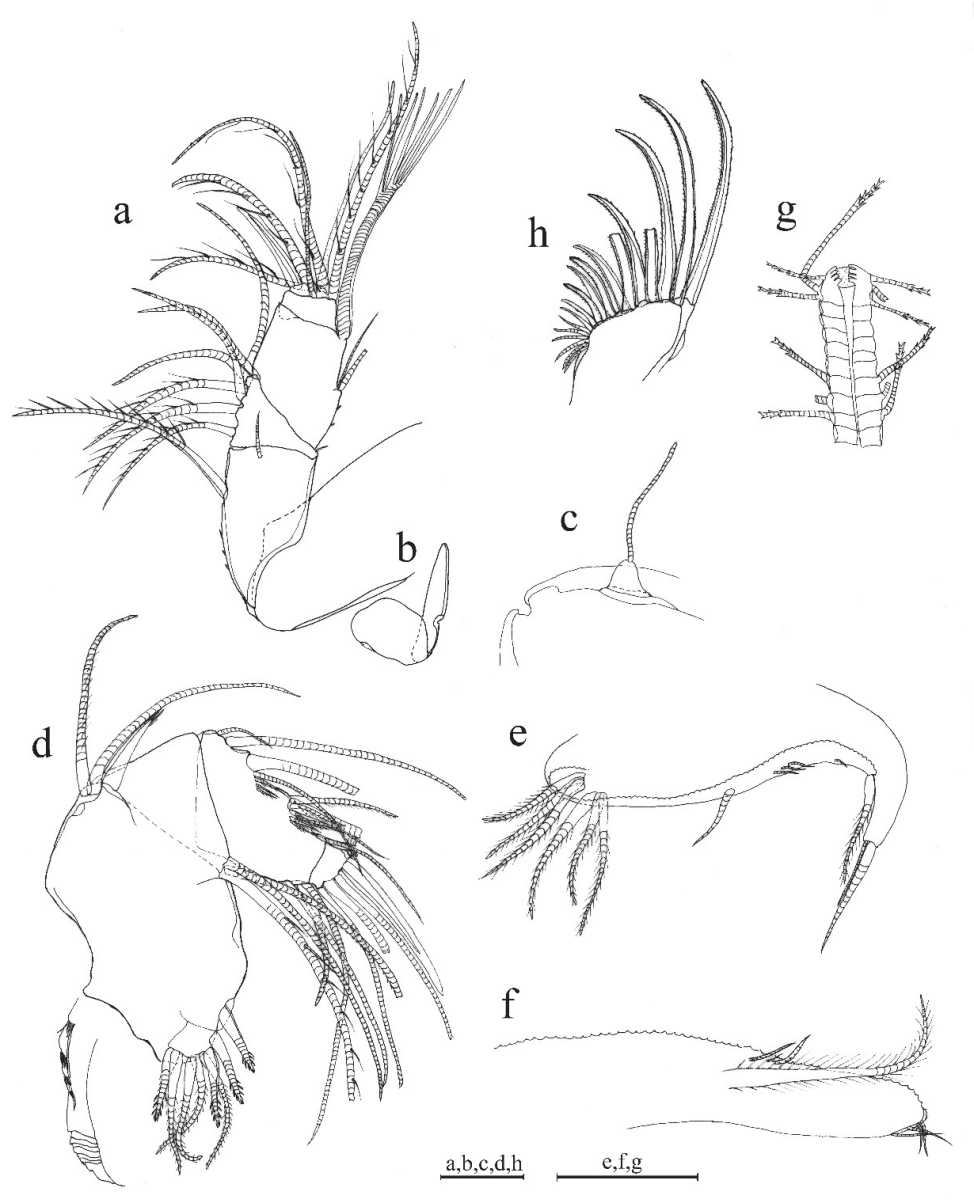
*Parasteropebusanensis* sp. nov. holotype female adult: **a**A1**b** Bellonci organ **c**A2**d**Md**e**Mxl**f**L5**g**L7**h** F. Scale bar: 0.1 mm.

A1 (Fig. 3a). 1^st^ joint: with no hair on broad surface; longer than 2^nd^ joint. 2^nd^ joint: one spinous dorsal seta, one short lateral bristle, and three proximal dorsal spines. 3^rd^ joint: seven setae (one short ventral and six dorsal). 4^th^ joint: three bristles (one long dorsal and two short ventral bristles) and two small ventral spines at mid length. 5^th^ joint: sensory bristle with six filaments without short proximal terminal. 6^th^ joint: one medial seta, attached to bottom of 7^th^ joint boundary. 7^th^ joint: a-bristle claw-like, bare; b-seta with marginal filaments; c-seta with marginal filaments. 8^th^ joint: d-bristle absent; e-bristle with blunt tip; f- and g-bristles with marginal filaments.

Bellonci organ (Fig. 3b). Elongate with rounded tip, constriction at mid-point.

***Eyes***. Lateral eye with many ommatidia obscured by black pigment; 13 ommatidia visible. Medial eye rounded and unpigmented.

A2. Protopodite: rounded without medial bristle. Endopodite (Fig. 3c): 2-jointed with long terminal bristle. Exopodite: 9-jointed; bristle of 2^nd^ segment with spinules along ventral margin and spines along dorsal margin reaching 8^th^ joint; 3^rd^–8^th^ joints with natatory hairs and spines along proximal part of ventral margin. 9^th^ article: four bristles (two long natatory and two short bristles). Articles 1–8 with minute spines at inner terminal corner.

Md (Fig. 3d). Coxale endite ventral branch with spines forming four oblique rows. Basale endite with four spinous end bristles, three triaenid bristles with three or four pairs spines excluding terminal pair. Basale dorsal margin with two long terminal setae, without mid-bristle. Exopodite with hirsute tip and two small subterminal setae, exopodite almost same length as dorsal margin of first endopodite article. Endopodite: 1^st^ joint: ventral margin with three bristles (one broken with short spines, one long with long marginal spines, and one shorter without spine or hair); 2^nd^ joint: ventral margin: with three terminal bristles; dorsal margin: with stout a-, b-, c-, and d-bristles and one short bristle proximal to a-bristle; one long e-bristle and three medial bristles forming an oblique row between b- and c-bristles and adjacent to b-bristle; four medial bristles forming an oblique row adjacent to c-bristle; and one short bristle adjacent to d-bristle; f-bristle between c- and d-bristles, long, bare; g-bristle longer than f-bristle. End segment with a strong dorsal claw-like bristle without marginal spines, three juxtaposed stout bristles of equal length, and two thin bristles.

Mxl (Fig. 3e). Endite I with three bristles. Endite II with three bristles. Basale: one bare proximal ventral bristle at the middle and four short distal ventral bristles. Endopodite: 1^st^ article absent α-seta and one hairy β-seta; 2^nd^ article with two terminal bristles.

L5 (Fig. 3f). Ventral section with fan of long setae. Comb: spinous exopodite bristle over past distal end of comb, two short bristles (ventral to base of exopodite bristle) with bases almost on ventral edge of comb, four bristles just proximal at the ventral edge of comb, without bristle to base of exopodite bristle.

L6. Unknown

L7 (Fig. 3g). Each limb with 12 bristles. Six proximal and six distal bristles (three on each side).

F (Fig. 3h). Each lamella with six claws and three posterior claws bristle-like. A total of nine claws and bristles.

**Male.** All features are comparable to adult female; important differences are listed below.

Shell (Fig. 2g–i). Carapace more elongate than that of female and with more open incisure, hairs forming vertical row near posterior end, carapace length 1.36 mm, height at middle 0.9 mm.

**Figure 4.**
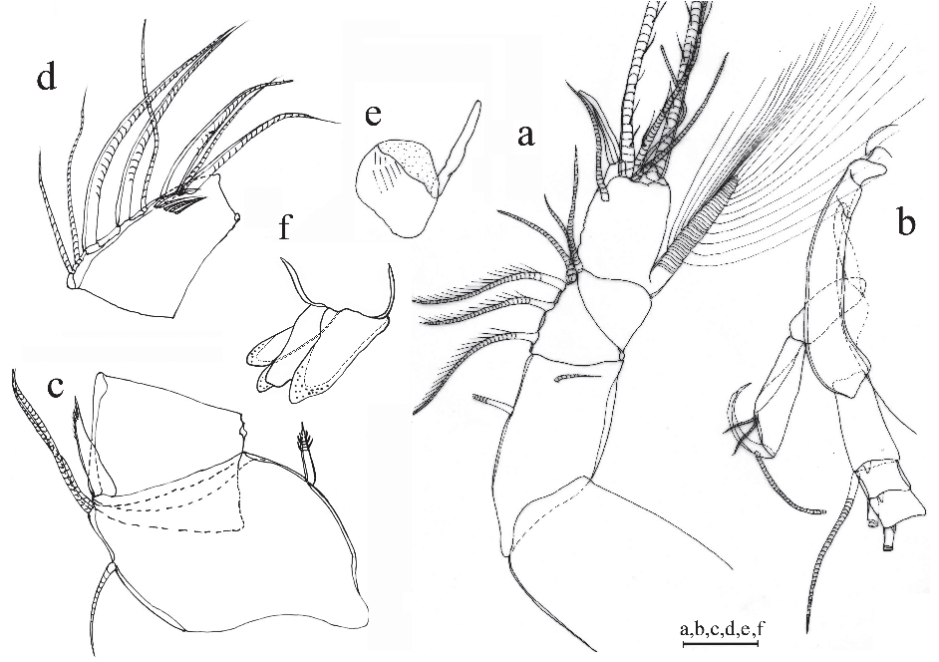
*Parasteropebusanensis* sp. nov. paratype male adult: **a**A1**b**A2**c**Md**d** Bellonci organ. Scale bar: 0.1 mm.

A1 (Fig. 4a). Sensory seta with robust stem and many filaments. 7^th^ and 8^th^ joints with very long c- and f- setae (long as carapace length) each with numerous of marginal filaments. 2^nd^ and 4^th^ joints without dorsal margin spines.

A2 (Fig. 4b). Endopodite with 3-jointed, article two with two lateral setae, article three recurved with 1 proximal seta.

Md (Fig. 4c). Basale dorsal margin with one seta at mid-length. Endopodite: two relatively long bristles proximal to a-bristle.

***Eyes*** (Fig. 4d). Lateral eye with more than 17 ommatidia. Medial eye rounded and unpigmented.

***Reproductive organs*** (Fig. 4f). Copulatory limbs are coalesced proximally, in generally, shorter than the furca lamella. The lobe is distally subdivided into three small lobes.

##### Remarks.

The new species is closely related to *P.iota* Kornicker, Harrison-Nelson & Coles, 2007, described from Kapua Channel (Waikïkï, Hawaii). The first joint of the A1 of *P.busanensis* is without hair on the broad surface, present in *P.iota*. In addition, the 3^rd^–8^th^ joints of *P.iota* each have fairly stout proximal ventral spines, absent in *P.busanensis*. The distal margin of the Mxl of *P.busanensis* bears four bristles compared to two in *P.iota*, and three bristles (one minute) near the ventral margin rather than five in *P.iota*. The new species shares many characters with *P.gamurru* Syme & Poore, 2006 described from Queensland, Australia (Syme and Poore 2006a), but lacks the nodes on the dorsal margin of the 5^th^ joint on the A1, which is a prominent character of *P.gamurru*. The new species also bears two oblique rows of cleaning setae on the medial side of the Md 2^nd^ joint, compared to only one in *P.gamurru*. The endite II of *P.busanensis* differs from that of *P.pacifica* Kornicker and Harrison-Nelson, 2005 from Johnston Atoll (Kornicker and Harrison-Nelson 2005) in having three rather than four bristles. The 3^rd^ joint of the Md in *P.theta* Kornicker, Harrison-Nelson & Coles, 2007 from Waikïkï, Hawaii (Kornicker et al. 2007). The same character is present in *P.sigma* Kornicker, Harrison-Nelson & Coles, 2007 from Käneohe Bay, Hawaii (Kornicker et al. 2007), and *P.zeta* Kornicker, 1986 from the Gulf of Mexico (Kornicker 1986).

The sexual dimorphism found in the new species, the morphology of the dorsal mid-bristle on the basale of Md, and the number of bristles proximal to the a-bristle on the endopodite of the Md, have never been described in *Parasterope*.

GenBank numbers 16S: OK048681, OK048682; 18S: OK048719, OK048720.

#### ﻿ Parasterope sagami sp. nov.

Taxon classificationAnimaliaMyodocopidaCylindroleberididae

1236E572-6871-5BD4-8A4E-A7A868F027CA

http://zoobank.org/5404E848-CCDC-4025-8E9F-B3725DCADD8A

Figs 5 , 6 , 7

##### Specimens examined.

***Holotype*** female dissected on one slide, shells on SEM stub (NIBR IV0000890541). ***Paratypes***: one male and one female dissected on separate slides, shells on SEM stub (NIBR IV0000890542). All from the type locality: Japan, Kanagawa, Sagami Bay (35°09.420'N, 139°36.556'E), 5 m, collected during 6^th^JAMBIO project on 13 February 2015.

##### Etymology.

The species name was chosen after the Sagami Bay, from where the species was collected. It is an adjective agreeing with female gender of the genus.

##### Diagnosis.

Surface of the shell completely smooth. Posterior infold of the carapace with a broad shelf. Dorsal margin rounded in female and almost perpendicular in male. Lateral eye well developed with black pigmented ommatidia, smaller in female than in male. A1 8-segmented without d-bristle on the 8^th^ joint. Uropodal lamellae with eight claws.

##### Description.

**Figure 5.**
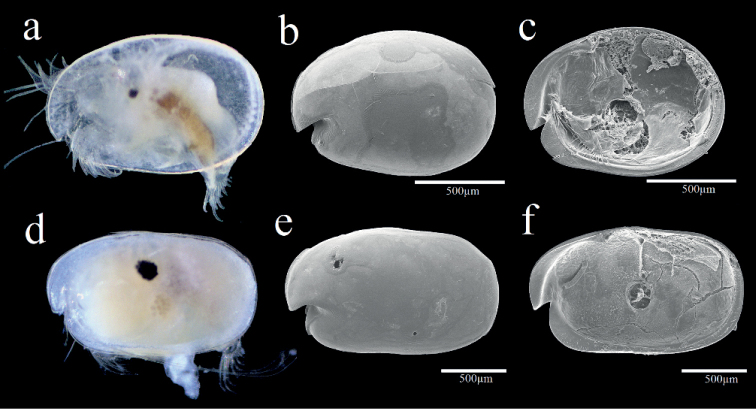
Light microscopy and scanning electron microscopy images of *Parasteropesagami* sp. nov. **a–c** holotype female adult: **a** External view **b** Lateral view from right valve **c** Internal view from left valve. **d–f** paratype male adult: **d** External view **e** Lateral view from right valve **f** Internal view of left valve.

**Female.** Shell (Fig. 5a–c). Carapace oval, broadening at posterior, greatest height near middle, carapace length 1.21 mm, height 0.83 mm. Carapace smooth and without ornamentation.

**Figure 6.**
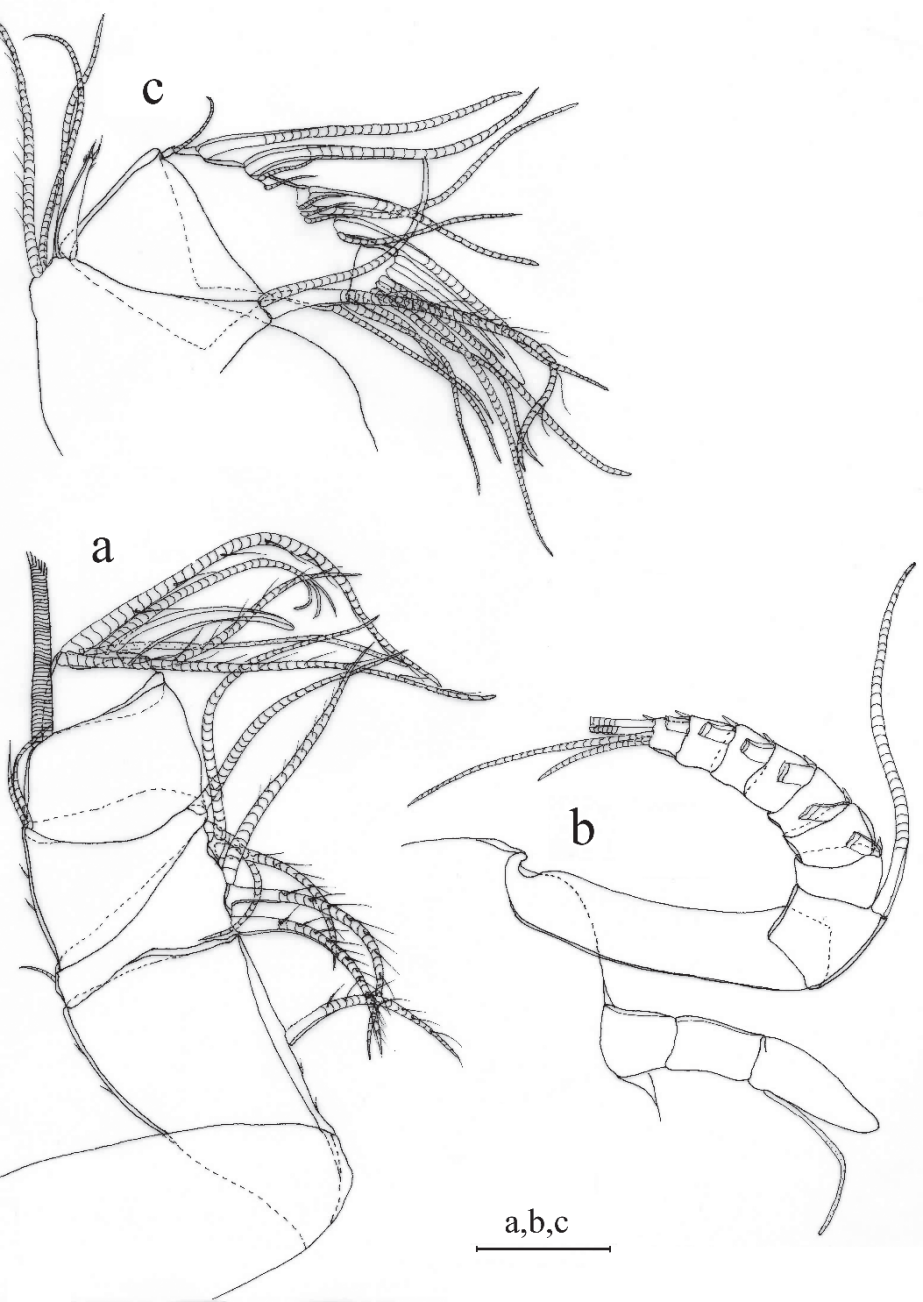
*Parasteropesagami* sp. nov. holotype female adult: **a**A1**b**A2**c**Md. Scale bar: 0.1 mm.

A1 (Fig. 6a). 1^st^ joint: with no hair on broad surface. 2^nd^ joint: one spinous dorsal seta, no lateral bristle, and two dorsal spines on each side of dorsal margin. 3^rd^ joint: seven setae: one short ventral and six dorsal. 4^th^ joint: three setae (one long dorsal bristle and two short ventral bristle) and two small ventral spines. 5^th^ joint: sensory bristle with six filaments without short proximal terminal. 6^th^ joint: unidentified. 7^th^ joint: a-bristle claw-like, bare; b-seta with marginal filaments; c-seta with marginal filaments. 8^th^ joint: d-bristle absent; e-bristle bare, with blunt tip; f- bristle with marginal filaments; g-bristle with three filaments without proximal and marginal filament.

**Figure 7.**
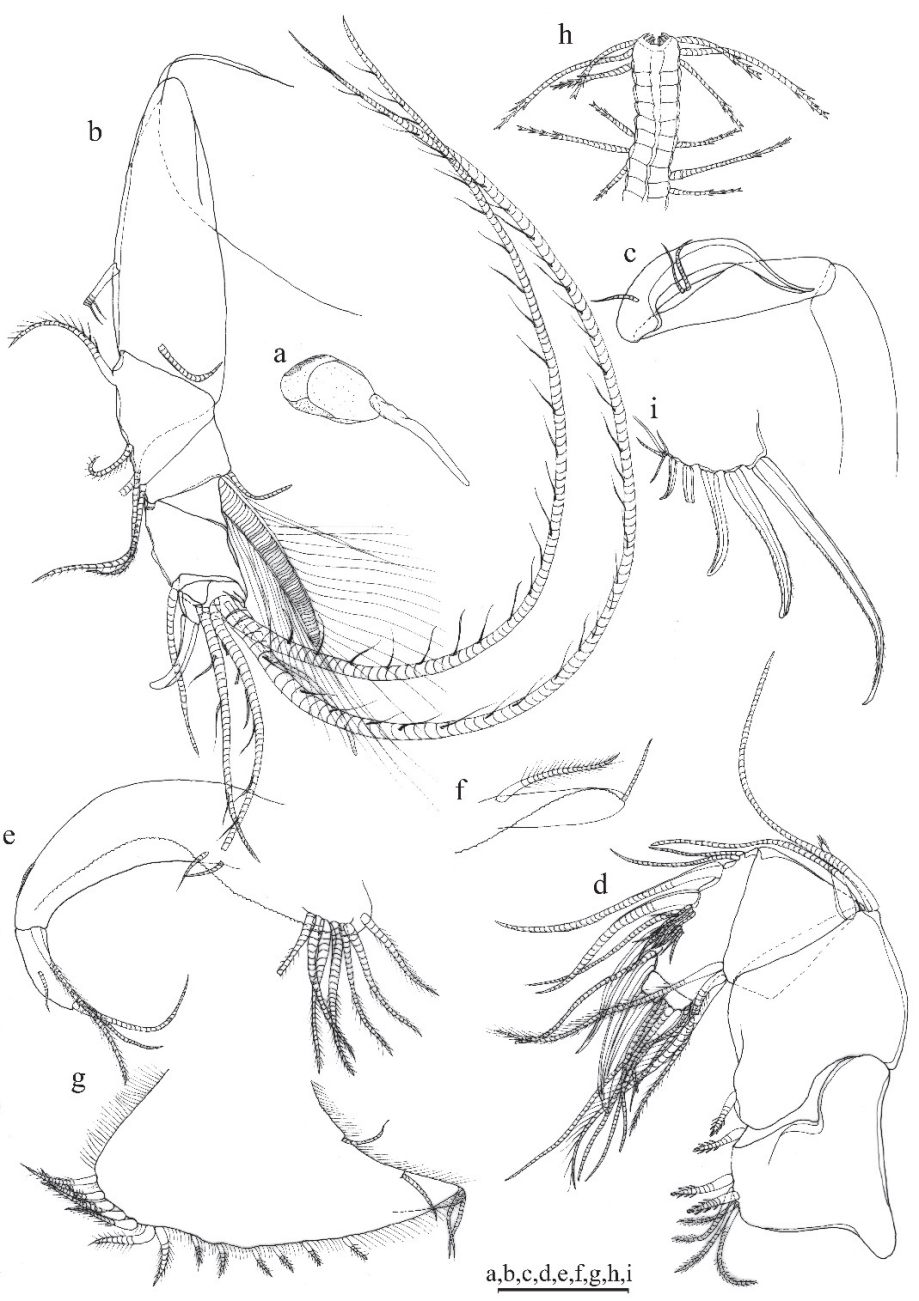
*Parasteropesagami* sp. nov. holotype male adult: **a** Bellonci organ **e**Mxl**f**L5**g**L6**h**L7**i** F. Paratype male adult: **b**A1**c**A2 endopodite **d**Md. Scale bar: 0.1 mm.

Bellonci orange (Fig. 7a). Elongate with rounded tip, unclear constriction.

***Eyes*** (Fig. 7a). Lateral eye with 12 ommatidia obscured by black pigment; Medial eye rounded and pigmented.

A2 (Fig. 6b). Protopodite: rounded without medial bristle. Endopodite: 3-jointed with long terminal bristle near junction between 2^nd^ and 3^rd^ join. Exopodite: 9-jointed; bristle of 2^nd^ segment along ventral margin and spines along dorsal margin reaching 8^th^ joint; 3^rd^–8^th^ joints with natatory hairs and spines along proximal part of ventral margin. 9^th^ article: four bristles (two long natatory and two short bristles). 1^st^–8^th^ with minute spines at inner terminal corner.

Md (Fig. 6c). Coxale endite: unable to determine. Basale endite with four spinous end bristles, three triaenid bristles with three pairs spines excluding terminal pair. Basale dorsal margin with three long terminal setae, without mid-bristle. Exopodite with hirsute tip and two small subterminal setae, exopodite length ~ ¾ of dorsal margin of first endopodite article. Endopodite: 1^st^ joint: ventral margin with three bristles (one missing, one long with long marginal spines, and one shorter without spine or hair); 2^nd^ joint: ventral margin: with three terminal bristles; dorsal margin: with stout a-, b-, c-, and d-bristles and one short bristle proximal to a-bristle; e-bristle missing, two medial bristles adjacent to b-bristle; three medial bristles forming an oblique row adjacent to c-bristle; f-bristle between c- and d-bristle, long, bare; g-bristle shorter than f-bristle. End segment with a strong dorsal claw-like bristle without marginal spines, three juxtaposed stout bristles of equal length.

Mxl (Fig. 7e). Endite I with three bristles. Endite II with four bristles. Basale: one dorsal medial distal bristle, two ventral medial proximal bristles. Endopodite: 1^st^ with one short α-seta and one hairy β-seta; 2^nd^ article with two terminal bristles.

L5 (Fig. 7f). Ventral section with fan of long setae. Comb: spinous exopodite bristle over past distal end of comb, one bristle just proximal at the ventral edge of comb.

L6 (Fig. 7g). Anterior margin with one endite bristle. Ventral margin with two spinous anterior bristles separated by space from 13 bristles. Anterior, ventral, and posterior margins, and medial surfaces hirsute. Lateral flap spinous but without bristles.

L7 (Fig. 7h). Each limb with 12 bristles. Six proximal and six distal bristles (three on each side).

F (Fig. 7i). Each lamella with five claws and three posterior claws bristle-like. A total of eight claws and bristles.

**Male.** All features comparable to adult female; important differences are:

Shell (Fig. 5d–f). Carapace more elongate than that of female and, carapace length 1.65 mm, height at middle 1.02 mm.

A1 (Fig. 7b). Sensory seta with robust stem and many filaments. 7^th^ and 8^th^ joints with very long c- and f-setae (long as carapace length) each with numerous of marginal filaments. 2^nd^ joint with lateral bristle. 2^nd^ and 4^th^ joints without dorsal margin spines.

***Eyes*.** Lateral eye with 18 ommatidia obscured by black pigment.

A2 (Fig. 7c). Endopodite with three articles, article two with two lateral setae, article three recurved with one small proximal seta.

Md (Fig. 7d). Basale dorsal margin with two long terminal setae. Endopodite: 2^nd^ joint: dorsal margin: with stout a-, b-bristles, c-and d-claws bristle-like, and two short bristles proximal to a-bristle; e-bristle present on male. Two oblique rows between b- and c-bristles (a row adjacent to b-bristle with four bristles, a row adjacent to c-claw bristle-like with six bristles).

***Reproductive organs*.** Unknown.

##### Remarks.

*Parasteropesagami* differs from all other *Parasterope* representatives by the combination of the following characters:

**Female.** Dorsal margin of the Md basale has three long terminal setae next to the exopodite, and it has no mid-bristle. In addition, the A1 g-bristle has a peculiar shape, and there is no lateral bristle on the A1 2^nd^ joint;

**Male.** The presence of the c- and d-claws bristle-like on the Md endopodite 2^nd^ joint in the adult males is unique for the new species.

In addition to the above characters, *P.sagami* differs from *P.busanensis* and the morphology of the endite II of Mxl. Namely, the endite carries three rather than four bristles. Also, the short α-seta on the Mxl exopodite is absent in *P.busanensis*.

*Parasteropejenseni* was also described from Sagami Sea, Japan (Poulsen 1965) and it also has no lateral bristle on the A1 2^nd^ joint. It differs from *P.sagami* by having small lateral eyes with ~ 10 ommatidia; a long stem of the sensory bristle (~ 4 × the length of the 6^th^ joint); and the presence of spines on the ventral margin of the protopodite of A2 (Poulsen 1965).

*Parasteropeobesa* Poulsen, 1965 from Misaki, Japan has a lateral bristle on the 2^nd^ joint of A1 which is absent in the two above species; 4^th^ joint of the A1 is without lateral spines on the ventral margin otherwise present in the new species; and there are more than ten bristles on the ventral edge of the comb.

GenBank numbers 16S: OK048683, OK048684; 18S: OK048721, OK048722.

#### ﻿ Parasterope singula sp. nov.

Taxon classificationAnimaliaMyodocopidaCylindroleberididae

B608A858-1A05-53D3-BC12-BFA8435D5C36

http://zoobank.org/4C34D735-E105-40E7-83A4-67BA3BD2C9EF

Figs 8 , 9

##### Specimens examined.

***Holotype*** female dissected on one slide, shells on SEM stub (NIBR IV 0000887835). ***Paratypes***: two females dissected on one slide each, shells on SEM stub (NIBR IV 0000879835_2&3). The sample was collected from the type locality: South Korea, Chuja Island by Ho Young Soh on 29 November 2012.

##### Etymology.

The name is a Latin noun, *singula*, because only a female has been collected. The name is in nominative, feminine singular, agreeing in gender with the genus.

##### Diagnosis.

Surface of the shell completely smooth. Anterior end with a deep incisure and posterior infold of the carapace with a broad shelf. Posterior end wider than anterior. Dorsal margin rounded. Lateral eye well developed with black pigmented ommatidia. Uropodal lamellae with nine claws.

##### Description.

**Figure 8.**
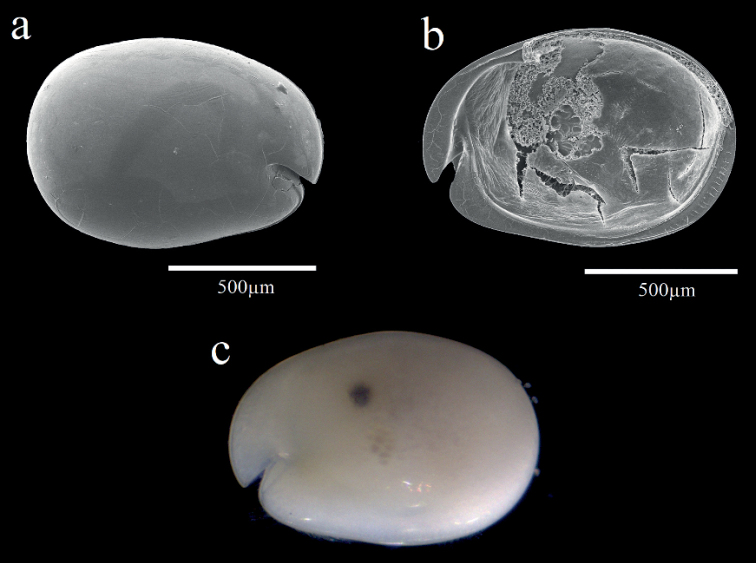
Light microscopy and scanning electron microscopy images of *Parasteropesingula* sp. nov. holotype female adult: **a** Lateral view from left valve **b** Internal view from left valve **c** External view.

**Female.** Shell (Fig. 8). Carapace oval, broadening at posterior, greatest height near middle, carapace length 1.25 mm, height 0.9 mm. Carapace smooth and without ornamentation.

A1 (Fig. 8a). 1^st^ joint: with no hair on broad surface. 2^nd^ joint: one spinous dorsal seta, one short lateral bristle, and small proximal dorsal spines. 3^rd^ joint: seven setae (one short ventral and six dorsal). 4^th^ joint: three bristles (one long dorsal bristle and two short ventral bristles). 5^th^ joint: sensory bristle with six filaments without short proximal terminal. 6^th^ joint: one medial seta, attached to bottom of 7^th^ joint boundary. 7^th^ joint: a-bristle claw-like, bare; b-seta with marginal filaments; c-seta with marginal filaments. 8^th^ joint: d-bristle absent; e-bristle bare with blunt tip; f- and g-bristles with marginal filaments.

**Figure 9.**
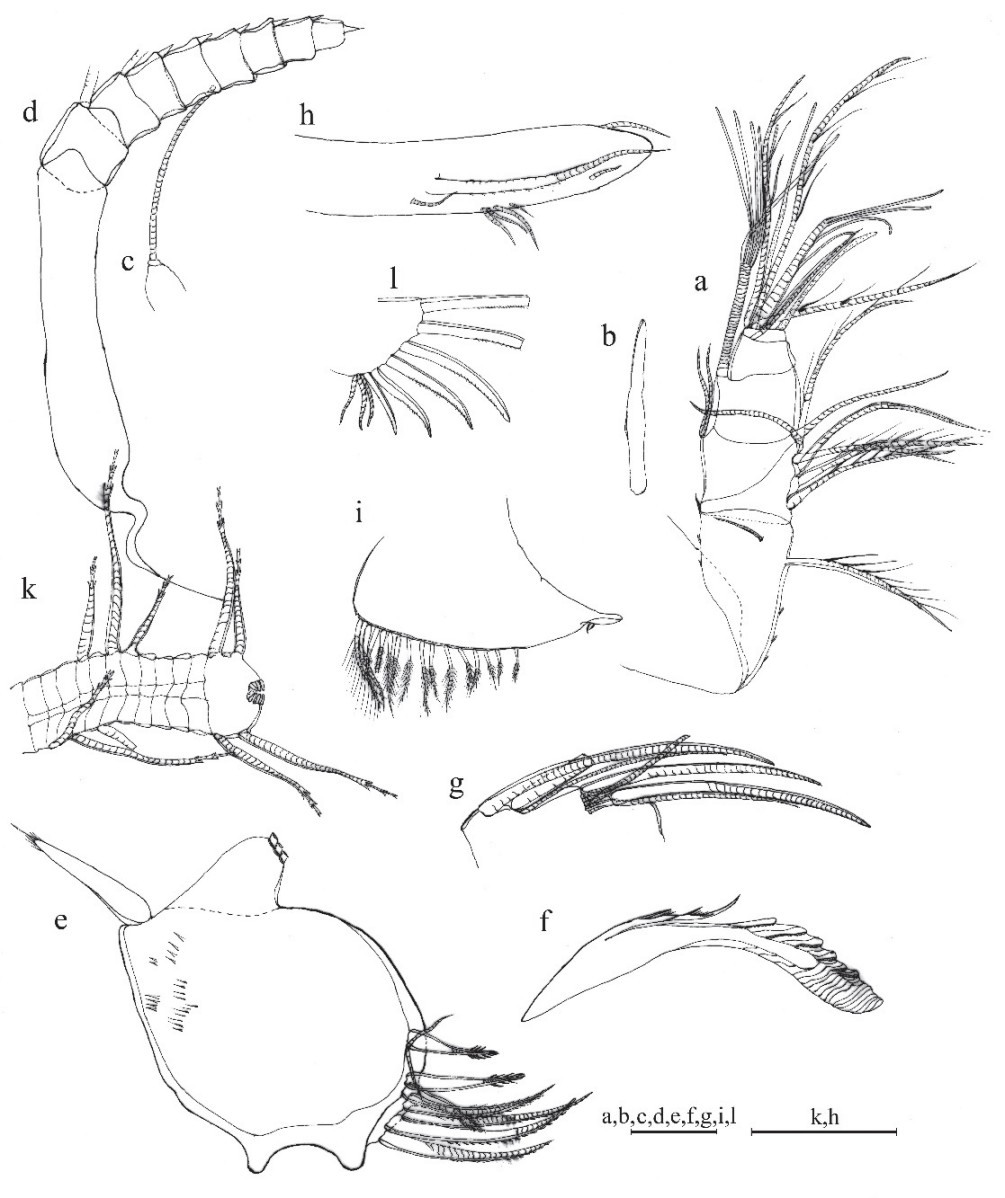
*Parasteropesingula* sp. nov. holotype female adult: **a**A1**b** bellonci organ **c**A2 endopodite **d**A2 exopodite **e**Md basale and coxale endite **f**Md basale endite **g**Md endopodite **h**L5**i**L6**k**L7**l** F. Scale bar: 0.1 mm.

Bellonci orange (Fig. 9b). Elongate with rounded tip with unclear constriction.

***Eyes*.** Lateral eye with 18 ommatidia.

A2 (Fig. 9c, d). Protopodite: rounded without medial bristle. Endopodite (Fig. 9c): not strongly jointed, with long terminal bristle. Exopodite (Fig. 9d): 9-jointed; bristle of 2^nd^ segment along ventral margin and spines along dorsal margin reaching 8^th^ joint; 3^rd^–8^th^ joints with natatory hairs and spines along proximal part of ventral margin. 9^th^ article: four bristles (two long natatory and two short bristles). 4^th^–8^th^ joints with minute spines at inner terminal corner.

Md (Fig. 9e–g). Coxale endite (Fig. 9e) same as that on *P.busanensis*. Basale (Fig. 9f): Basale endite with three spinous end bristles, four triaenid bristles with three spines excluding terminal pair, and two equal-length bare bristles. Basale dorsal margin with two long terminal setae, without mid-bristle; five rows of small spines on the broad surface near dorsal margin. Exopodite with hirsute tip and two small subterminal setae, exopodite almost same length as dorsal margin of first endopodite article. Endopodite (Fig. 9g): 1^st^ joint: ventral margin with three bristles (two long with long marginal spines, and one shorter without spine or hair); 2^nd^ joint: ventral margin: with three terminal bristles; dorsal margin: with stout a-, b-, c-, and d-bristles and without bristle proximal to a-bristle; one long e-bristle between b- and c-bristles; four medial bristles forming an oblique row adjacent to c-bristle; f-bristle between c- and d-bristles, long, bare; g-bristle longer than f-bristle. 3^rd^ segment with a strong dorsal claw-like bristle without marginal spines, three juxtaposed stout bristles of equal length, and two thin bristles.

Mxl. Endite I with three bristles. Endite II with three bristles. Basale: one dorsal medial distal bristle, two ventral medial proximal bristles. Endopodite: 1^st^ with one short α-seta and one hairy β-seta; 2^nd^ article with two terminal bristles.

L5 (Fig. 9h). Ventral section with fan of long setae. Comb with spinous exopodal bristle just reaching past distal end of comb, one short slender bristle just ventral to base of stout bristle, one bristle near exopodal bristle stem, and four lateral bristles set back from edge at comb mid length.

L6 (Fig. 9i). Unidentified number of endite bristle on the anterior margin. Lateral flap with hairs but no bristles. Anterior tip of skirt with two small bristles. Ventral and postero-ventral margin with 17 plumose bristles (one missing).

L7 (Fig. 9k). Each limb with ten bristles. Four proximal and six distal bristles.

F (Fig. 9l). Each lamella with six claws plus three posterior claws bristle-like. A total of nine claws and bristles.

**Male.** Not collected.

##### Remarks.

The presence of rows of small spines on the broad surface near the dorsal margin of the Md basale is a unique feature of *Parasteropesingula*. Additionally, the number of bristles on the L7 (ten bristles: four on the terminal segment (two on each side), six on the proximal segments (three on each side) is less than in all other *Parasterope* representatives. The new species is similar to *P.mckenziei*, described from Samar Province, Philippines (McKenzie, 1970) but differs in the following characters: the Md endopodite is without a proximal bristle next to the a-bristle, a character not known in any other *Parasterope*, and the number of claws on UL (nine vs. seven).

The 2^nd^ joint of the Md endopodite in *P.singula* carries one oblique row of cleaning setae, also recorded in *P.antyx* Kornicker, 1989 from Bay of Biscay (Kornicker, 1989), *P.gamma* Kornicker, Harrison-Nelson & Coles, 2007 from Hawaiian Islands (Kornicker et al. 2007), *P.hulingsi* Baker, 1978 from California (Baker 1978), *P.jenseni* from Sagami Sea, Japan (Poulsen 1965), *P.lagunicola* Hartmann, 1984 from French Polynesia (Hartmann 1984), *P.mckenziei* from the Philippines (Kornicker 1970), *P.muelleri* Skogsberg, 1920 from England (Poulsen 1965), *P.prolixa* Kornicker, 1975 from Australia (Kornicker 1975), *P.sequex* Kornicker & Poore, 1996 from Australia (Kornicker and Poore 1996), *P.styx* from Chile (Kornicker 1975), and *P.theta* from Waikïkï (Kornicker et al. 2007). The three other new species, *P.busanensis*, *P.sagami*, and *P.sohi* also have two oblique rows. The exopodite of the A2 in *P.singula* and *P.sohi* carries minute spines at inner terminal corner on the 5^th^–8^th^ joints, while these spines are present from the 1^st^–8^th^ joints in *P.busanensis* and *P.sagami*.

GenBank numbers 16S: OK048686, OK048687; 18S OK048723.

#### ﻿ Parasterope sohi sp. nov.

Taxon classificationAnimaliaMyodocopidaCylindroleberididae

1419F8FA-9E29-5D88-958E-A7676BDB9122

http://zoobank.org/94451EAB-B789-4279-9CB7-585F59753729

Figs 10 , 11

##### Specimens examined.

***Holotype*** female dissected on one slide, shells on SEM stub (NIBR IV 0000887834). The sample was collected from the type locality: South Korea, Maemul Island, 34°32'00.4"N, 128°43'54.4"E, by Ho Young Soh on 25^th^ July 2011.

##### Etymology.

The species named after Prof. Ho-young Soh (Faculty of Marin Technology, Chonam National University, Jeonnam, Korea), whom we are greatly indebted to for collecting and providing samples for this publication.

##### Diagnosis.

Carapace elongated. Surface of the shell completely smooth. Posterior end with a short, rounded incisure, while the anterior has relatively deep incisure. Dorsal and ventral margins rounded. Posterior end wider than anterior. Lateral eye well developed with black pigmented ommatidia.

##### Description.

**Figure 10.**
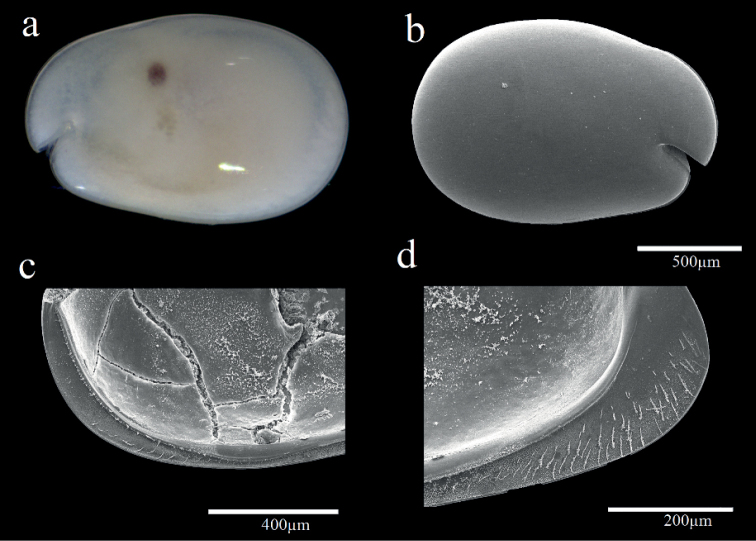
Light microscopy and scanning electron microscopy images of *Parasteropesohi* sp. nov. holotype female adult: **a** External view **b** Lateral view from left valve **c** Anterior of left valve from inside showing bristles of infold.

**Female.** Shell (Fig. 10). Carapace oval, broadening at posterior, greatest height near middle, carapace length 1.48 mm, height 0.98 mm. Carapace smooth and without ornamentation.

**Figure 11.**
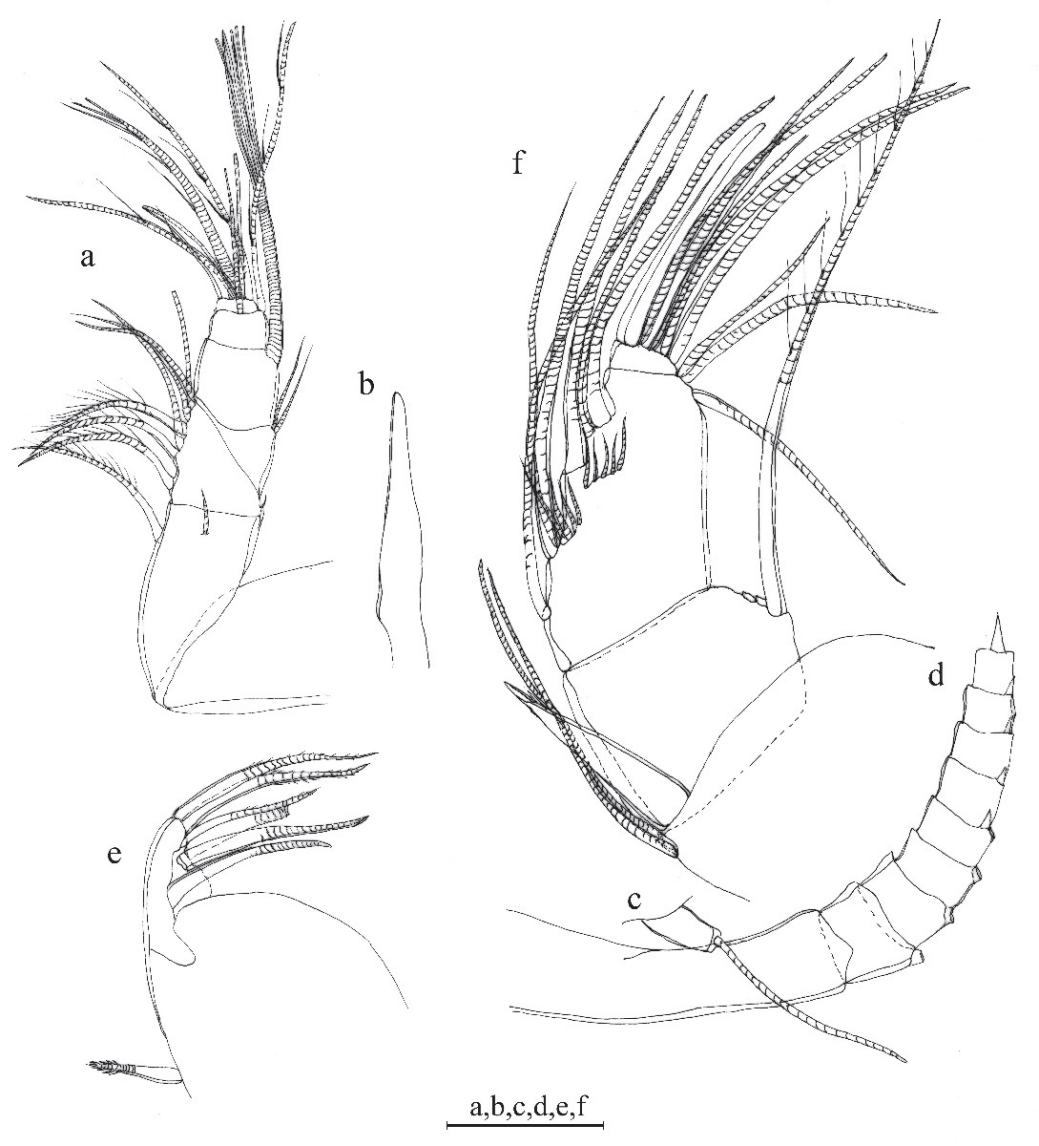
*Parasteropesohi* sp. nov. holotype female adult: **a**A1**b** bellonci organe **c**A2 endopodite **d**A2 exopodite **e**Md basale endite **f**Md endopodite. Scale bar: 0.1 mm.

A1 (Fig. 11a). 1^st^ joint: with no hair on broad surface and longer than 2^nd^ joint. 2^nd^ joint without spines along dorsal or ventral margin, one spinous dorsal seta, one short lateral bristle. 3^rd^ joint: seven setae (one short ventral and six dorsal). 4^th^ joint: three bristles (one long dorsal and two short ventral bristles). 5^th^ joint: sensory bristle with six filaments without short proximal terminal. 6^th^ joint: one medial seta, attached to bottom of 7^th^ joint boundary. 7^th^ joint: a-bristle claw-like, bare; b-seta with marginal filaments; c-seta with marginal filaments. 8^th^ joint: d-bristle absent; e-bristle bare, with blunt tip; f- and g-bristles with marginal filaments.

Bellonci orange (Fig. 11b). Elongate with rounded tip with unclear constriction.

***Eyes*.** Lateral eye with 15 ommatidia.

A2 (Fig. 11c, d). Protopodite: rounded. Endopodite (Fig. 11c): 2-jointed, with long terminal bristle, ~ 1/3 of stem length. Exopodite (Fig. 11d): 9-jointed; bristle of 2^nd^ segment along ventral margin and spines along dorsal margin reaching 8^th^ joint; 3^th^–8^th^ joints with natatory hairs and spines along proximal part of ventral margin. 9^th^ article: four bristles (two long natatory and two short bristles). 5^th^–8^th^ joints with minute spines at inner terminal corner.

Md (Fig. 11e, f). Coxale endite same as that on *P.busanensis* and *P.singula*. Basal endite (Fig. 11e) with six spinous end bristles and one triaenid bristle with three spines excluding terminal pair. Basale dorsal margin with two long terminal setae, without mid-bristle. Exopodite with spine-like tip and small subterminal setae, exopodite just reaching past end of dorsal margin of the 1^st^ endopodite article. Endopodite (Fig. 11f): 1^st^ joint: ventral margin with three long bristles with long marginal spines; 2^nd^ joint: ventral margin: with three terminal bristles; dorsal margin: with stout a-, b-, c-, and d-bristles and with one short bristle proximal to a-bristle (missing on drawing line figure); one long e-bristle between b- and c-bristles; four medial cleaning bristles forming an oblique adjacent to b-bristle; five medial bristles forming an oblique row adjacent to c-bristle; f-bristle between c- and d-bristles, long, bare; g-bristle longer than f-bristle. 3^rd^ segment with a strong dorsal claw-like bristle without marginal spines, three juxtaposed stout bristles of equal length, and two thin bristles.

Mxl. Endite unknown. Basale: one dorsal medial distal bristle. Endopodite: 1^st^ with one short α-seta and one hairy β-seta; 2^nd^ article with two terminal bristles.

L5. Unknown.

L6. Unknown.

L7. Unknown.

F. Unknown.

**Male.** Not collected.

##### Remarks.

The new species *Parasteropesohi* sp. nov. is poorly known because of the missing L5, L6, L7, and furca but we propose a new name because of the presence of the combination of the following characters: A1 sensory bristle carry 0+6 filaments; exopodite of Md is unusually long; and the Md endopodite has the e-bristle. In majority of *Parasterope* described so far, the mandible exopodite is at most longer than ½ length of the dorsal margin of the 1^st^ endopodite joint; in *P.sohi* the exopodite length is greater than the length of the dorsal margin of the 1^st^ endopodite joint. Other species with such a long exopodite include *P.lagunicola* from French Polynesia (Hartmann 1984), *P.omega* Kornicker, Harrison-Nelson & Coles, 2007 from Hawaiian Islands (Kornicker, Harrison-Nelson and Coles 2007), and *P.sohni* Kornicker & Caraion, 1974 from West Africa (Kornicker and Caraion 1974). However, the first two species have small proximal spines on A1, absent in *P.sohi.* The last species has a mid-bristle on the dorsal margin of the basale, absent in *P.sohi*. Also, *P.sohi* differs from another *Parasterope* species by having six spinous end bristles and one triaenid bristle on the endite of the Md basale. All other *Parasterope* species are armed with at least four triaenid bristles.

GenBank number 16S: OK048685. 18S could not be obtained.

### ﻿A checklist of species of *Parasterope*

A checklist of species the 49 named *Parasterope*, including four new species described in this study, is presented in Suppl. material 1. This is an updated species checklist published by Syme and Poore (2006b), and at the World Ostracoda Database (Brandão et al. 2022).

## ﻿Results of molecular analysis

**Figure 12.**
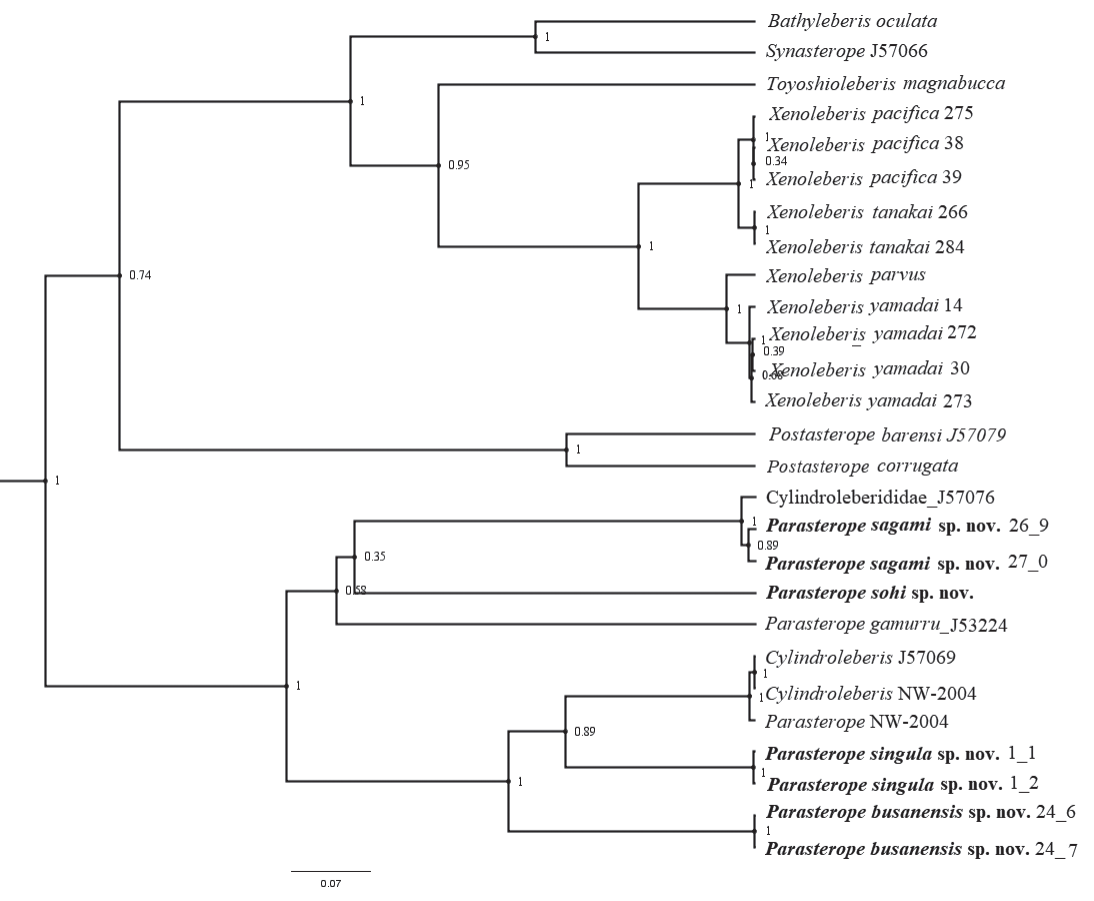
Bayesian inference cladogram of the Cylindroleberidinae subfamily based on 16S rRNA sequences. Numbers at nodes represent posterior probability.

The final alignment of the 16S data set consisted of 33 sequences 445 base pairs long, of which 210 were constant sites (= 47.191% of all sites), 210 were invariant (= 47.191% of all sites), 207 were parsimony informative, and 275 were distinct site patterns. The substitution model TPM2+F+I+G4 was found to be the best fit evolutionary model. Effective sample size for all the continuous parameters (posterior, prior, tree likelihood, tree height, Yule model, and birthrate) estimated by Tracer analysis was far above a recommended number (200). Phylogram is presented on the Fig. 12. The results of pairwise distance analysis between species used in this study are shown in Suppl. material 2: Table S1. The interspecies pairwise distances between 16S rRNA sequences belonging to *Parasterope* ranged between 22% (between *Parasterope* sp. NW-2004 and *P.sohi*) and 45% (between *P.sohi* and *P.busanensis*). The pairwise distance between Parasterope and the other Cylindroleberidinae groups varied between 0% (with *Cylindroleberis*) and 59% (with *X.yamadai*). Distances between genera (Suppl. material 3: Table S2) varied from 32.7% (between *Cylindroleberis* and *Bathyleberis*) to 53.3% (between *Postasterope* and *Parasterope*). Of all genera included in the analyses, *Parasterope* had the highest values of intrageneric distances of 32.2%. We failed to amplify 18S sequence of *P.sohi*, however, distance between 18S of other *Parasterope*, as well as distance between Cylindroleberidinae genera, for which data are available, are presented in the Suppl. material 4: Table S3. Distances between genera were less than 4% (in the range between 0.3% and 3.7%). Similar values were calculated for the intrageneric distances (from 1.0% to 4.0%). Interspecies pairwise distances between 18S sequence are shown in the Suppl. material 5: Table S4. The highest value was 4% between *Cylindoleberis* sp. J57069 and *C.marranyin*.

The resulting phylogenetic tree based on the newly obtained sequences and those belonging to the subfamily Cylindroleberidinae reconstructed based on the 16S alignment (Fig. 12) suggests that *Parasterope* is not a monophyletic taxon, as it clusters with several *Cylindroleberis* sequences, downloaded from GenBank. *Parasterope* sequences, *Cylindroleberis* sequences, and one unidentified sequence from GenBank form a clade separat from the rest of the included genera. Two of the species described from Korea, *P.busanensis* and *P.singula* form a monlophyletic unit together with two undescribed *Cylindroleberis* and one *Parasterope* species downloaded from GenBank. This branch received a high posterior probability support of 1. The Japanese species, *P.sagami* clusters with one GenBank sequences attributed to an unidentified Cylindroleberididae taxon, and the branch has a high posterior probability support. The third Korean species, *P.sohi* clustered with *P.gamurru* and the new Japanese species, but this branch did not receive a high posterior probability support.

## ﻿Discussion

The unusual clustering of *Parasterope* and several unidentified Cylindroleberididae and *Cylindroleberis* on the phylogenetic tree, assigned to other genera, should be considered as misidentification and/or contaminations, and not an indication of polyphyletic nature of *Parasterope.* This can be supported by the results from previous, more complex, phylogenetic analyses of the entire family Cylindroleberididae (Syme and Oakley 2012; Pham et al. 2021) where the same GenBank sequences have been used, but they did not cluster with *Parasterope* sequences.

Due to a very limited number of available sequences attributed to the subfamily Cylindroleberidinae (27 in total) our phylogenetic tree included only 18 species belonging to seven genera. This can be considered a very poor sample, since the subfamily contains ~ 200 species belonging to 23 genera (see Pham et al. 2021; Brandão et al. 2022). Therefore, the interpretation of our results is very limited. However, it should be noted that only two out of three newly described Korean species form a monophyletic unit, while the third species, *P.busanensis* clusters with the Japanese species *P.sagami*, albeit with almost no support.

The results of pairwise distance analysis show a fast evolutionary rate of 16S rRNA, in contrary to 18S rRNA. Calculated pairwise distances between 18S sequences were very similar to those of other for Cylindroleberidae, Cypridinidae, and Philomedidae published by Pham et al. (2020). These results indicate that 18S gene marker does not provide enough resolution for the reconstruction of phylogenetic relationships below the subfamily level. The pairwise distance values of this genetic marker between genera in the subfamily Cylindroleberidinae mainly ranges from 1 to 2%, and even < 1% in the case of *Synasterope* and *Postasterope* (0.2%), indicating that 18S is ineffective genetic tools to construct a phylogenetic tree at a lower taxonomic level (below family) of this target taxa. From these results and those published in previous work (Pham et al. 2020; Pham et al. 2021), we recommend using a combination of fast-evolving gene markers (COI, 16S) and highly-conservative gene markers (18S and 28S) for constructing a phylogenetic tree for the Myodocopida group rather than analysis based on a single marker.

In the Syme and Oakley (2012) study where molecular and morphological data for the entire family Cylindroleberididae were combined to study phylogenetic relationships, *Parasterope* was polyphyletic. This may be due to the fact that molecular data set was smaller than morphological data set for all included genera. Nevertheless, it is very likely that the new genetic data will change current view on the phylogeny of this diverse genus, and its position within the subfamily.

In terms of biodiversity, our results are important as they provide new data for the marine fauna of Korea and Japan. They also point out that samples taken from areas where human impact on ecosystems is high due to dense population and industrial development can yield new species, and enrich our knowledge on the biodiversity of the planet.

## Supplementary Material

XML Treatment for Parasterope busanensis

XML Treatment for Parasterope sagami

XML Treatment for Parasterope singula

XML Treatment for Parasterope sohi

## Supplementary materials

Supplementary material 1 A checklist species of *Parasterope* Kornicker, 1975 Data type Checklist Explanation note A checklist of species of *Parasterope* Kornicker, 1975. https://binary.pensoft.net/file/672101This dataset is made available under the Open Database License (http://opendatacommons.org/licenses/odbl/1.0/). The Open Database License (ODbL) is a license agreement intended to allow users to freely share, modify, and use this Dataset while maintaining this same freedom for others, provided that the original source and author(s) are credited.Huyen T. M. Pham, Ivana Karanovic

Supplementary material 2 Table S1 Data type gene distances Explanation note Pairwise p-distances among 16S sequences of species presented in Fig. 12. https://binary.pensoft.net/file/672102This dataset is made available under the Open Database License (http://opendatacommons.org/licenses/odbl/1.0/). The Open Database License (ODbL) is a license agreement intended to allow users to freely share, modify, and use this Dataset while maintaining this same freedom for others, provided that the original source and author(s) are credited.Huyen T. M. Pham, Ivana Karanovic

Supplementary material 3 Table S2 Data type gene distances Explanation note Between group means distance of 16S sequences from Cylindroleberidinae genera. IG: intrageneric. https://binary.pensoft.net/file/672103This dataset is made available under the Open Database License (http://opendatacommons.org/licenses/odbl/1.0/). The Open Database License (ODbL) is a license agreement intended to allow users to freely share, modify, and use this Dataset while maintaining this same freedom for others, provided that the original source and author(s) are credited.Huyen T. M. Pham, Ivana Karanovic

Supplementary material 4 Table S3 Data type gene distances Explanation note Between group means distance of 18S sequences from Cylindroleberidinae genera. IG: intrageneric. https://binary.pensoft.net/file/672104This dataset is made available under the Open Database License (http://opendatacommons.org/licenses/odbl/1.0/). The Open Database License (ODbL) is a license agreement intended to allow users to freely share, modify, and use this Dataset while maintaining this same freedom for others, provided that the original source and author(s) are credited.Huyen T. M. Pham, Ivana Karanovic

Supplementary material 5 Table S4 Data type gene distances Explanation note Pairwise p-distances among 18S sequences of species presented in Table 1. https://binary.pensoft.net/file/672105This dataset is made available under the Open Database License (http://opendatacommons.org/licenses/odbl/1.0/). The Open Database License (ODbL) is a license agreement intended to allow users to freely share, modify, and use this Dataset while maintaining this same freedom for others, provided that the original source and author(s) are credited.Huyen T. M. Pham, Ivana Karanovic
