# Supplementary material for: ﻿Four new Parasterope (Ostracoda, Myodocopina) from the Northwest Pacific and their phylogeny based on 16S rRNA
Source: Zookeys. 2022 Apr 13;1095:13–42. doi: 10.3897/zookeys.1095.77996 (PMC9021157; doi:10.3897/zookeys.1095.77996)
Supplement: Supplementary material 1 — A checklist species of Parasterope Kornicker, 1975 [file zookeys-1095-013-s001.docx]

**A checklist species of *Parasterope* Kornicker, 1975**

**Parasterope Kornicker, 1975**

*Parasterope* Poulsen, 1965: 361 [nomen nudum]

*Parasterope* Kornicker, 1975: 401 [designated type species]

Type species. *Asterope mulleri* Skogsberg, 1920 by original designation, Kornicker, 1975

1. ***Parasterope aberrata* Skogsberg, 1920**

*Asterope aberrata* Skogsberg, 1920: 504

*Parasterope aberrata* Poulsen, 1965: 362

Type locality. Strangford Lough, Ireland

1. ***Parasterope alpha* Kornicker & Caraion, 1974**

*Parasterope alpha* Kornicker & Caraion, 1974: 11

Type locality. Mauritania, 560 m depth, 18°40’00N, 16°41’05’’W

1. ***Parasterope anommata*** **Kornicker, 1975**

*Parasterope anommata* Kornicker, 1975: 427

Type locality. Continental slope off Point Descado, east of Argentina, 424-428 m depth

1. ***Parasterope antyx*** **Kornicker, 1989**

*Parasterope antyx* Kornicker, 1989: 97

Type locality. West of Bay of Biscay, 1560 m depth, 47°39’N, 8°11’48’’W

1. ***Parasterope australis* (Brady, 1890)**

*Astrope australis* Brady, 1898: 431

*Asterope australis* Scott, 1912: 431

*Parasterope australis* Eagar, 1971: 61

Type localitySuYa, inside reef; Mango Island, fringing reef; Apia, Upolu, reef and shore pools, Noumea, New Caledonia, dredged, 2-4 fathoms

1. ***Parasterope beta* Kornicker, 1976**

*Parasterope beta* Kornicker, 1976: 11

Type locality. Luderitz Bay, Namibia, south-west Africa

1. ***Parasterope busanesis* sp. nov.**

*Parasterope busanesis* sp. nov., this study

Type locality. Busan, Min-rack harbor, South Korea, 35°09'11.9"N 129°07'38.4"E

1. ***Parasterope crinita* Kornicker, 1975**

*Parasterope crinita* Kornicker, 1975: 421

Type locality. Subantarctic, east of South Island, New Zealand, 51

1. ***Parasterope extrachelata* Kornicker, 1958**

*Asteropina extrachelata* Kornicker, 1958: 241

*Synasterope extrachelata* Poulsen, 1965: 355

*Parasterope extrachelata* Kornicker, 1986: 28

Type locality. Cavelle Pond, salt-water pond on South Bimini, Great Bahama Bank

1. ***Parasterope gamma* Kornicker, Harrison-Nelson & Coles, 2007**

*Parasterope gamma* Kornicker, Harrison-Nelson & Coles, 2007: 60

Type locality. Station 10, 22 Jan 2001, Kapua Channel, Waikïkï, O‘ahu, Hawaiian Islands

1. ***Parasterope gamurru* Syme & Poore, 2006**

*Parasterope gamurru* Syme & Poore, 2006: 52

Type locality. Patch Reef, Lizard Island, Queensland, Australia, 3 m, 14°41.038' S, 145°27.003' E

1. ***Parasterope hirutai* Chavtur, 1983**

*Parasterope hirutai* Chavtur, 1983: 73, 76

Type locality. Japan, 504 m, 39°59'N 142°19'3"E

1. ***Parasterope hulingsi* Baker, 1978**

*Cylindroleberis psitticina* Darby, 1965 : 317

*Parasterope hulingsi* Baker, 1978: 145

Type locality. South of San Mateo Point, California, 13.7 m , 33°17’35’’N, 117°28’50’’W

1. ***Parasterope iota* Kornicker, Harrison-Nelson & Coles, 2007**

*Parasterope iota* Kornicker, Harrison-Nelson & Coles, 2007: 86

Type locality. Sta 10, 22 Jan 2001, Kapua Channel, Waikïkï, Hawaiian Islands

1. ***Parasterope jenseni* Poulsen, 1965**

*Parasterope jenseni* Poulsen, 1965: 387

Type locality. Okinose, Sagami Sea, Japan, 180 m depth

1. ***Parasterope kappa* Kornicker, 1976**

*Parasterope kappa* Kornicker, 1976: 18

Type locality. Luderitz Bay, Namibia, south-west Africa

1. ***Parasterope lagunicola* Hartmann, 1984**

*Parasterope lagunicola* Hartmann, 1984: 121

Type locality. Kalkalgen vom Anleger der Kia Ora Village Lagune

1. ***Parasterope longiseta* Skogsberg, 1920**

*Asterope mulleri* var. *longiseta* Skogsberg, 1920: 467

*Parasterope muelleri longiseta* Poulsen, 1965: 469

*Parasterope longiseta* Kornicker, 1975: 405

Type locality. Port William, Falkland Islands, 71 m depth

1. ***Parasterope longungues* Poulsen, 1965**

*Parasterope longungues* Poulsen, 1965: 382

Type locality. Off Frederickssted, St. Croix, Virgin Islands, 900 m

1. ***Parasterope lux* Kornicker in Kornicker & Poore, 1996**

*Parasterope lux* Kornicker, in Kornicker & Poore, 1996: 110

Type locality. South of Point Hicks, Victoria, Australia, 1000 m depth. 38°21.90’S, 149°20.00’E

1. ***Parasterope maddocksae* Kornicker in Kornicker & Thomassin, 1998**

*Parasterope maddocksae* Kornicker & Thomassin, 1998: 117

Type locality. Tulear Reef Complex, Grand Recif, southwest Madagascar, 24 m depth

1. ***Parasterope mauritania* Kornicker & Caraion, 1974**

*Parasterope mauritania* Kornicker & Caraion, 1974: 18

Type locality. Mauritania, 52 m depth, 20°10’05’’N, 17°32’09’’W

1. ***Parasterope mckenziei* Kornicker, 1970**

*Parasterope mckenziei* Kornicker, 1970: 26

Type locality. Southwest of Botic Island, Salcedo, Samar Province, Philippines, 1 m depth, 11°05’N, 125°41’E

1. ***Parasterope micrommata* Kornicker, 1975**

*Parasterope micrommata* Kornicker, 1975: 419

Type locality. Subantarctic, near Macquarie Island, 112 m depth

1. ***Parasterope muelleri* Skogsberg, 1920**

*Cypridina teres* Norman, 1861

*Asterope teres* Müller 1912: 46 [part]

*Asterope mulleri* Skogsberg 1920: 483

*Asteropina muelleri* Kornicker, 1958: 239

*Parasterope muelleri* Poulsen, 1965: 370

Type locality. English Channel, off Salcombe, coast of England

1. ***Parasterope nana* Poulsen, 1965**

*Parasterope nana* Poulsen, 1965: 396

Type locality. Koh Kam, Thailand, 18 m depth

1. ***Parasterope obesa* Poulsen, 1965**

*Parasterope obesa* Poulsen, 1965: 364

Type locality. Misaki, Japan, shallow water

1. ***Parasterope ohlini* Skogsberg, 1920**

*Asterope ohlini* Skogsberg, 1920: 467

*Parasterope ohlini* Poulsen1965: 363

*Parasterope lowryi* Kornicker, 1971: 197

Non *Parasterope lowryi* (Skogsberg): Lofthouse, 1967: 143

Type locality. South Georgia, off Grytviken, Antarctic, 24-52 m depth, 54°22’S, 36°27’W

Taxonomic discussion on synonymy: see Kornicker (1975: 2)

1. ***Parasterope omega* Kornicker, Harrison-Nelson & Coles, 2007**

*Parasterope omega* Kornicker, Harrison-Nelson & Coles, 2007: 65

Type locality. Sta 6, 19 Nov 1999, Pu‘u Kaua‘i reef, Käne‘ohe Bay, O‘ahu, Hawaiian Islands

1. ***Parasterope pacifica* Kornicker & Harrison-Nelson, 2005**

*Parasterope pacifica* Kornicker & Harrison-Nelson, 2005: 325

Type locality. Johnston Atoll, north of Akau Island, 2-3.5 m depth, 16°45’53.3’’N, 169°31’2.4’’W

1. ***Parasterope pectinata* Poulsen, 1965**

*Parasterope pectinata* Poulsen, 1965: 367

Type locality. Three Kings Island, northern New Zealand, 100 m depth

1. ***Parasterope physinx* Kornicker in Kornicker & Poore, 1996**

*Parasterope physinx* Kornicker, in Kornicker & Poore, 1996: 112

Type locality. South of Point Hicks, Victoria, Australia, 400 m depth, 38°17.70’S, 149°11.30’E

1. ***Parasterope pollex* Kornicker in Bowman & Kornicker, 1967**

*Parasterope pollex* Bowman & Kornicker, 1967: 9

*Parasterope* sp. indet. Kornicker, 1977: 794

Type locality. Hadley Harbor, Massachusetts

1. ***Parasterope prolixa* Kornicker, 1975**

*Parasterope prolixa* Kornicker, 1975: 436

Type locality. Subantarctic, Fortesque Bay, Strait of Magellan, 21 m depth

1. ***Parasterope pseudoquadrata* Hartmann** **in Hartmann-Schröder & Hartmann, 1965**

*Cylindroleberis pseudoquadrata* Hartmann, in Hartmann-Schröder & Hartmann, 1965: 316

*Parasterope pseudoquadrata* Kornicker, 1975: 415

Type locality. Bahia Ingles, North Chile, 12 m depth, 41°48’S, 73°53’W

1. ***Parasterope quadrata* Brady, 1898**

*Asterope quadrata* Brady, 1898: 432

Not *Synasterope quadrata* Poulsen, 1965: 406

*Synasterope quadrata* Eagar, 1971: 61 [part]

*Parasterope quadrata* Kornicker, 1975: 409

Type locality. Lyttelton Harbor, New Zealand, 2-10 m depth

1. ***Parasterope sagami* sp. nov.**

*Parasterope sagami* sp. nov., this study

Type locality. Kanagawa, Sagami Bay, Japan, 35°09.420'N 139°36.556'E

1. ***Parasterope sequax* Kornicker in Kornicker & Poore, 1996**

*Parasterope sequax* Kornicker, in Kornicker & Poore, 1996: 106

Type locality. South of Point Hicks, Victoria, Australia, 1500 m depth. 38°25.00’S, 149°00.00’E

1. ***Parasterope sigma* Kornicker, Harrison-Nelson & Coles, 2007**

*Parasterope sigma* Kornicker, Harrison-Nelson & Coles, 2007: 70

Type-locality. Station 1, 17 Nov 1999, North Channel, Käne‘ohe Bay, Hawaiian Islands

1. ***Parasterope singula* sp. nov.**

*Parasterope singula* sp. nov., this study

Type locality. Chuja Island, South Korea

1. ***Parasterope skogsbergi* Poulsen, 1965**

*Parasterope skogsbergi* Poulsen, 1965: 380

Type locality. Koh Kahdat, Thailand, 2 m.

1. ***Parasterope sohi* sp. nov.**

*Parasterope sohi* sp. nov., this study

Type locality. Maemul Island, South Korea, 34°32'00.4"N 128°43'54.4"E

1. ***Parasterope sohni* Kornicker & Caraion, 1974**

*Parasterope sohni* Kornicker & Caraion, 1974: 14

Type locality. West Africa, 534 m depth, 21°47’00’’N, 17°36’06’’W

1. ***Parasterope styx* Kornicker, 1975**

*Parasterope styx* Kornicker, 1975: 432

Type locality. South Pacific, west of Chile, 4303 m, 37°57’S, 75°08’W

1. ***Parasterope theta* Kornicker, Harrison-Nelson & Coles, 2007**

*Parasterope theta* Kornicker, Harrison-Nelson & Coles, 2007: 80

Type Locality. Sta 11, 22 Jan 2001, Kaiser’s Channel, Waikïkï, Hawaiian Islands

1. ***Parasterope thrix* Kornicker & Caraion, 1974**

*Parasterope thrix* Kornicker & Caraion, 1974: 21

Type locality. Mauritania, 270 m depth, 20°50’05’’N, 17°39’00’’W

1. ***Parasterope whatleyi* Kornicker in Kornicker & Poore, 1996**

*Parasterope whatleyi* Kornicker, in Kornicker & Poore, 1996: 115

Type locality. New South Wales, Australia, 996 m depth. 34°52.72’S, 151°15.04’E

1. ***Parasterope zamboangae* Kornicker, 1970**

*Parasterope zamboangae* Kornicker, 1970

*Parasterope zamboangae* Kornicker, 1970: 23

Type locality. Sacol Island, Zamboanga Province, Philippines, 1–2 m depth

1. ***Parasterope zeta* Kornicker, 1986**

*Parasterope zeta* Kornicker, 1986: 47

Type locality. Gulf of Mexico, off Galveston, Texas, 58 m depth

References

Baker JH (1978) Two new species of *Parasterope* (Myodocopina, Ostracoda) from southern California. Crustaceana 35(2): 139–151. <https://doi.org/10.1163/156854078X00042>

Bowman TE, Kornicker LS (1967) Two new crustaceans: The parasitic copepod *Sphaeronellopsis monothrix* (Choniostomatidae) and its myodocopid ostracod host *Parasterope pollex* (Cylindroleberididae) from the Southern New England coast. Proceedings of the United States National Museum 123(3613): 1–30. <https://doi.org/10.5479/si.00963801.123-3613.1>

Brady GS (1890) On Ostracoda collected by H.B. Brady, Esq., L.L.D., F.R.S. in the South Sea Islands. Transactions of the Royal Society of Edinburgh. Earth Sciences 35(2): 489–525. <https://doi.org/10.1017/S0080456800017749>

Brady GS (1898) On new or imperfectly known species of Ostracoda, chiefly from New Zealand. Transactions of the Zoological Society of London 14: 489–525.

Chavtur VG (1983) Ostrakody Myodocopina, Cladocopina umerennykh I kholodnykh vod Severnogo polusharila. [Ostracodes (Myodocopina, Cladocopina) of Temperate and Cold Waters of the Northern Hemisphere.]. Academy of Sciences of the USSR, Far-Eastern Science Center, Institute of Marine Biology, 132 pp.

Darby DG (1965) Ecology and taxonomy of Ostracoda in the vicinity of Sapelo Island, Georgia. Kesling, R.V. (ed.) Four Reports of Ostracod Investigations. Ann Arbor, University of Michigan, Michigan: 1–77.

Eagar SH (1971) A check list of the Ostracoda of New Zealand. Journal of the Royal Society of New Zealand 1: 53–64. <https://doi.org/10.1080/03036758.1971.10419355>

Hartmann G (1984) Zur Kenntnis der Ostracoden der polynesischen Inseln Huahiné (Gesellschaftsinseln) und Rangiroa (Tuamotu-Inseln). Mit Bemerkungen zur Verbreitung und Ausbreitung litoraler Ostracoden und einer Übersicht über die bislang auf den pazifischen Inseln gefunden. Mitteilungen aus dem Hamburgischen Zoologischen Museum und Institut 81: 117–169.

Hartmann-Schröder G, Hartmann G (1965) Zur Kenntnis des Sublitorals der chilenischen Küste unter besonderer Berücksichtigung der Polychaeten und Ostracoden. Mitteilungen aus dem Hamburgischen Zoologischen Museum und Institut 62: 1–384.

Kornicker LS (1958) Ecology and taxonomy of recent marine Ostracodes in the Bimini Area, Great Bahama Bank. Publications of the Institute of Marine Science 5: 194–300.

Kornicker LS (1970) Myodocopid Ostracoda (Cypridinacea) from the Philippine Islands. Smithsonian Contributions to Zoology 39: 1–32. <https://doi.org/10.5479/si.00810282.39>

Kornicker LS (1971) Benthic Ostracoda (Myodocopina: Cypridinacea) from the South Shetland Islands and the Palmer Archipelago, Antarctica. Antarctic Research Series 17: 167–216.

Kornicker LS (1975) Antarctic Ostracoda (Myodocopina), Part 2. Smithsonian Contributions to Zoology 163: 375–720. https://doi.org/10.5479/si.00810282.163

Kornicker LS (1976) Myodocopid Ostracoda from Southern Africa. Smithsonian Contributions to Zoology 214: 1–39.

Kornicker LS (1986) Cylindroleberididae of the Western North Atlantic and the Northern Gulf of Mexico, and Zoogeography of the Myodocopina (Ostracoda). Smithsonian Contributions to Zoology 425: 1–139. <https://doi.org/10.5479/si.00810282.425>

Kornicker LS (1989) Bathyal and Abyssal Myodocopid Ostracoda of the Bay of Biscay and Vicinity. Smithsonian Contributions to Zoology 467: 1–134. <https://doi.org/10.5479/si.00810282.467>

Kornicker LS, Caraion FE (1974) West African Myodocopid Ostracoda (Cylindroleberididae). Smithsonian Contributions to Zoology 179: 1–88.

Kornicker LS, Harrison-Nelson E (2005) Ostracoda from Johnston Atoll, Pacific Ocean, and proposal of a new tribe, Bruuniellini (Myodocopina: Cylindroleberididae). Pacific Science 59(3): 323–362. <https://doi.org/10.1353/psc.2005.0038>

Kornicker LS, Poore GC (1996) Ostracoda (Myodocopina) of the SE Australian Continental Slope: Part 3. Smithsonian Contributions to Zoology 573: 1–186. <https://doi.org/10.5479/si.00810282.573>

Kornicker LS, Thomassin BA (1998) Ostracoda (Myodocopina) of Tulear Reef Complex, SW Madagascar. Smithsonian Contributions to Zoology 595: 1–134.

Kornicker LS, Harrison-Nelson E, Coles SL (2007) Ostracoda (Myodocopina) from O‘ahu and French Frigate Shoals, Hawaiian Islands. Bishop Museum bulletin in Zoology 8: 1–128.

Müller GW (1912) Ostracoda. Das Tierreich, Crustacea 31. R. Friedländer und Sohn, Berlin, 1–428.

Norman AM (1861) Contributions to British Carcinology. Annals and Magazine of Natural History, Series 3 (8): 273–281.

Poulsen EM (1965) Ostracoda - Myodocopa, Part II: Cypridiniformes - Rutidermatidae, Sarsiellidae and Asteropidae. Dana Report Carlsberg Foundation (65): 1–484.

Skogsberg T (1920). Studies on marine ostracods. Part 1. (Cypridinids, Halocyprids and Polycopids). Zoologiska bidrag fran Uppsala, Supplement 1: 1–784.

Syme AE, Poore GCB (2006) Three new ostracod species from coastal Australian waters (Crustacea: Ostracoda: Myodocopa: Cylindroleberididae). Zootaxa 1305 (1): 51–67. <https://doi.org/10.11646/zootaxa.1305.1.5>
