# Supplementary material for: ﻿Four new Parasterope (Ostracoda, Myodocopina) from the Northwest Pacific and their phylogeny based on 16S rRNA
Source: Zookeys. 2022 Apr 13;1095:13–42. doi: 10.3897/zookeys.1095.77996 (PMC9021157; doi:10.3897/zookeys.1095.77996)
Supplement: Supplementary material 2 — Table S1 [file zookeys-1095-013-s002.docx]

| **Genus** | **Species** | **GB number** | |
| --- | --- | --- | --- |
|  |  | 16S | 18S |
| Bathyleberis | B. oculata | EU587251^1^ | EU591814^1^ |
|  | C. marranyin |  | EU587243^1^ |
| Cylindroleberis | Cylindroleberis J57069 | EU587253^1^ | EU587244^1^ |
|  | Cylindroleberis NW-2004 | AY624729^2^ |  |
| UnID | Cylindroleberididae J57076 | EU587257^1^ |  |
| Parasterope | P. busanensis sp. nov. 24_6 | **OK048681** | **OK048719** |
|  | P. busanensis sp. nov. 24_7 | **OK048682** | **OK048720** |
|  | P. gamurru J53224 | EU587255^1^ | EU591819^1^ |
|  | P. pollex |  | AF363309^4^ |
|  | P. sagami sp. nov. 26_9 | **OK048683** | **OK048721** |
|  | P. sagami sp. nov. 27_0 | **OK048684** | **OK048722** |
|  | P. singula sp. nov. 1_1 | **OK048686** | **OK048723** |
|  | P. singula sp. nov. 1_2 | **OK048687** |  |
|  | P. sohi sp. nov. | **OK048685** |  |
|  | P. styx |  | EU587236^1^ |
|  | Parasterope J57072* |  | EU587247^1^ |
|  | Parasterope NW-2004 | AY624728^2^ |  |
| Postasterope | P. barensi J57079 | EU587258^1^ | EU587248^1^ |
|  | P. corrugata | EU587259^1^ | EU591816^1^ |
| Synasterope | Synasterope J57066 | EU587252^1^ | EU587250^1^ |
|  | Synasterope J57067 |  | EU591815^1^ |
| *Toyoshioleberis* | *T. magnabucca* | MW534153^3^ | MZ092883^3^ |
| *Xenoleberis* | *X. parvus* | MW534150^3^ |  |
|  | *X. pacifica* 38 | MW534151^3^ | MZ092881^3^ |
|  | *X. pacifica* 39 | MW534152^3^ | MZ092882^3^ |
|  | *X. pacifica* 275 | MW534140^3^ | MZ092883^3^ |
|  | *X. tanakai* 266 | MW534141^3^ |  |
|  | *X. tanakai* 284 | MW534142^3^ | MZ092884^3^ |
|  | *X. yamadai* 272 | MW534143^3^ | MZ092879^3^ |
|  | *X. yamadai* 273 | MW534144^3^ | MZ092880^3^ |
|  | *X. yamadai* 14 | MW534145^3^ | MZ092878^3^ |
|  | *X. yamadai* 30 | MW534147^3^ |  |
