# Supplementary material for: ﻿Four new Parasterope (Ostracoda, Myodocopina) from the Northwest Pacific and their phylogeny based on 16S rRNA
Source: Zookeys. 2022 Apr 13;1095:13–42. doi: 10.3897/zookeys.1095.77996 (PMC9021157; doi:10.3897/zookeys.1095.77996)
Supplement: Supplementary material 3 — Table S2 [file zookeys-1095-013-s003.docx]

|  |  |  |  |  |  |  | **IG** |
| --- | --- | --- | --- | --- | --- | --- | --- |
| *Bathyleberis* |  |  |  |  |  |  | n/c |
| *Cylindroleberis* | 0.327 |  |  |  |  |  | 0.261 |
| *Parasterope* | 0.400 | 0.263 |  |  |  |  | 0.322 |
| *Postasterope* | 0.532 | 0.473 | 0.535 |  |  |  | 0.139 |
| *Synasterope* | 0.300 | 0.486 | 0.482 | 0.420 |  |  | n/c |
| *Toyoshioleberis* | 0.389 | 0.415 | 0.464 | 0.453 | 0.447 |  | n/c |
| *Xenoleberis* | 0.356 | 0.484 | 0.497 | 0.410 | 0.372 | 0.361 | 0.077 |
