# Supplementary material for: ﻿Four new Parasterope (Ostracoda, Myodocopina) from the Northwest Pacific and their phylogeny based on 16S rRNA
Source: Zookeys. 2022 Apr 13;1095:13–42. doi: 10.3897/zookeys.1095.77996 (PMC9021157; doi:10.3897/zookeys.1095.77996)
Supplement: Supplementary material 4 — Table S3 [file zookeys-1095-013-s004.docx]

|  |  |  |  |  |  |  | **IG** |
| --- | --- | --- | --- | --- | --- | --- | --- |
| *Bathyleberis* |  |  |  |  |  |  | n/c |
| *Cylindroleberis* | 0.037 |  |  |  |  |  | 0.047 |
| *Parasterope* | 0.020 | 0.007 |  |  |  |  | 0.013 |
| *Postasterope* | 0.015 | 0.003 | 0.005 |  |  |  | 0.003 |
| *Synasterope* | 0.017 | 0.003 | 0.004 | 0.002 |  |  | n/c |
| *Toyoshioleberis* | 0.020 | 0.013 | 0.014 | 0.012 | 0.010 |  | n/c |
| *Xenoleberis* | 0.020 | 0.013 | 0.014 | 0.012 | 0.010 | 0.010 | 0.000 |
