# Supplementary material for: ﻿Four new Parasterope (Ostracoda, Myodocopina) from the Northwest Pacific and their phylogeny based on 16S rRNA
Source: Zookeys. 2022 Apr 13;1095:13–42. doi: 10.3897/zookeys.1095.77996 (PMC9021157; doi:10.3897/zookeys.1095.77996)
Supplement: Supplementary material 5 — Table S4 [file zookeys-1095-013-s005.docx]

| *Bathyleberis oculata* |  |  |  |  |  |  |  |  |  |  |  |  |  |  |  |  |  |  |  |  |  |  |
| --- | --- | --- | --- | --- | --- | --- | --- | --- | --- | --- | --- | --- | --- | --- | --- | --- | --- | --- | --- | --- | --- | --- |
| *Cylindroleberis marranyin* | 0.03 |  |  |  |  |  |  |  |  |  |  |  |  |  |  |  |  |  |  |  |  |  |
| *Cylindroleberis* sp. J57069 | 0.04 | 0.04 |  |  |  |  |  |  |  |  |  |  |  |  |  |  |  |  |  |  |  |  |
| *Parasterope busanensis* sp nov 24_6 | 0.02 | 0.01 | 0.01 |  |  |  |  |  |  |  |  |  |  |  |  |  |  |  |  |  |  |  |
| *Parasterope busanensis* sp nov 24_7 | 0.02 | 0.01 | 0.01 | 0.00 |  |  |  |  |  |  |  |  |  |  |  |  |  |  |  |  |  |  |
| *Parasterope gamurru* J53224 | 0.02 | 0.01 | 0.01 | 0.00 | 0.00 |  |  |  |  |  |  |  |  |  |  |  |  |  |  |  |  |  |
| *Parasterope singula* sp. nov. 1_1 | 0.01 | 0.01 | 0.00 | 0.01 | 0.01 | 0.01 |  |  |  |  |  |  |  |  |  |  |  |  |  |  |  |  |
| *Parasterope pollex* | 0.03 | 0.01 | 0.01 | 0.01 | 0.01 | 0.01 | 0.01 |  |  |  |  |  |  |  |  |  |  |  |  |  |  |  |
| *Parasterope sagami* sp nov 26_9 | 0.02 | 0.01 | 0.01 | 0.00 | 0.00 | 0.00 | 0.01 | 0.01 |  |  |  |  |  |  |  |  |  |  |  |  |  |  |
| *Parasterope sagami* sp nov 27_0 | 0.02 | 0.01 | 0.01 | 0.00 | 0.00 | 0.00 | 0.01 | 0.01 | 0.00 |  |  |  |  |  |  |  |  |  |  |  |  |  |
| *Parasterope styx* | 0.02 | 0.01 | 0.01 | 0.00 | 0.00 | 0.00 | 0.01 | 0.01 | 0.00 | 0.00 |  |  |  |  |  |  |  |  |  |  |  |  |
| *Postasterope barensi* | 0.01 | 0.01 | 0.00 | 0.01 | 0.01 | 0.01 | 0.00 | 0.01 | 0.01 | 0.01 | 0.01 |  |  |  |  |  |  |  |  |  |  |  |
| *Postasterope corrugata* | 0.02 | 0.00 | 0.00 | 0.00 | 0.00 | 0.00 | 0.00 | 0.01 | 0.00 | 0.00 | 0.00 | 0.00 |  |  |  |  |  |  |  |  |  |  |
| *Synasterope* sp. J57066 | 0.02 | 0.00 | 0.00 | 0.00 | 0.00 | 0.00 | 0.00 | 0.01 | 0.00 | 0.00 | 0.00 | 0.00 | 0.00 |  |  |  |  |  |  |  |  |  |
| *Toyoshioleberis magnabucca* | 0.02 | 0.01 | 0.01 | 0.01 | 0.01 | 0.01 | 0.01 | 0.02 | 0.01 | 0.01 | 0.01 | 0.01 | 0.01 | 0.01 |  |  |  |  |  |  |  |  |
| *Xenoleberis pacifica* 275 | 0.02 | 0.01 | 0.01 | 0.01 | 0.01 | 0.01 | 0.01 | 0.02 | 0.01 | 0.01 | 0.01 | 0.01 | 0.01 | 0.01 | 0.00 |  |  |  |  |  |  |  |
| *Xenoleberis pacifica* 38 | 0.02 | 0.01 | 0.01 | 0.01 | 0.01 | 0.01 | 0.01 | 0.02 | 0.01 | 0.01 | 0.01 | 0.01 | 0.01 | 0.01 | 0.00 | 0.00 |  |  |  |  |  |  |
| *Xenoleberis pacifica* 39 | 0.02 | 0.01 | 0.01 | 0.01 | 0.01 | 0.01 | 0.01 | 0.02 | 0.01 | 0.01 | 0.01 | 0.01 | 0.01 | 0.01 | 0.00 | 0.00 | 0.00 |  |  |  |  |  |
| *Xenoleberis tanakai* 284 | 0.02 | 0.01 | 0.01 | 0.01 | 0.01 | 0.01 | 0.01 | 0.02 | 0.01 | 0.01 | 0.01 | 0.01 | 0.01 | 0.01 | 0.00 | 0.00 | 0.00 | 0.00 |  |  |  |  |
| *Xenoleberis yamadai* 14 | 0.02 | 0.01 | 0.01 | 0.01 | 0.01 | 0.01 | 0.01 | 0.02 | 0.01 | 0.01 | 0.01 | 0.01 | 0.01 | 0.01 | 0.00 | 0.00 | 0.00 | 0.00 | 0.00 |  |  |  |
| *Xenoleberis yamadai* 272 | 0.02 | 0.01 | 0.01 | 0.01 | 0.01 | 0.01 | 0.01 | 0.02 | 0.01 | 0.01 | 0.01 | 0.01 | 0.01 | 0.01 | 0.00 | 0.00 | 0.00 | 0.00 | 0.00 | 0.00 |  |  |
| *Xenoleberis yamadai* 273 | 0.02 | 0.01 | 0.01 | 0.01 | 0.01 | 0.01 | 0.01 | 0.02 | 0.01 | 0.01 | 0.01 | 0.01 | 0.01 | 0.01 | 0.00 | 0.00 | 0.00 | 0.00 | 0.00 | 0.00 | 0.00 |  |
| *Xenoleberis yamadai* 30 | 0.02 | 0.01 | 0.01 | 0.01 | 0.01 | 0.01 | 0.01 | 0.02 | 0.01 | 0.01 | 0.01 | 0.01 | 0.01 | 0.01 | 0.00 | 0.00 | 0.00 | 0.00 | 0.00 | 0.00 | 0.00 | 0.00 |
